# Active Zone Maturation Controls Presynaptic Output and Release Mode and Is Regulated by Neuronal Activity

**DOI:** 10.1523/JNEUROSCI.1143-25.2025

**Published:** 2025-10-14

**Authors:** Yulia Akbergenova, Jessica Matthias, Sofya Makeyeva, J. Troy Littleton

**Affiliations:** ^1^Departments of Brain and Cognitive Sciences, The Picower Institute for Learning and Memory, Massachusetts Institute of Technology, Cambridge, Massachusetts 02139; ^2^Biology, The Picower Institute for Learning and Memory, Massachusetts Institute of Technology, Cambridge, Massachusetts 02139; ^3^Abberior Instruments America, Bethesda, Maryland 20814

**Keywords:** active zone, *Drosophila*, neurotransmitter release, NMJ, synapse, synaptic vesicle fusion

## Abstract

Synapse formation requires the accumulation of cytomatrix proteins and voltage-gated Ca^2+^ channels (VGCCs) at presynaptic active zones (AZs). At *Drosophila melanogaster* larval neuromuscular junctions, a sequential process of AZ maturation is observed, with initial incorporation of early scaffolds followed by arrival of late scaffolds and VGCCs. To examine how AZ maturation regulates presynaptic output, serial imaging of AZ formation and function was performed at time-stamped synapses of male larvae expressing glutamate receptors linked to the photoconvertible protein mMaple. Quantal imaging demonstrated older synapses have higher synaptic efficacy and sustain greater release across development, while immature sites lacking VGCC accumulation supported spontaneous fusion. To examine how activity regulates AZ maturation, the effects of cell autonomous disruptions to neurotransmitter release were analyzed. Decreased synaptic transmission reduced AZ seeding and caused hyperaccumulation of material at existing AZs. Generation of an endogenous photoconvertible version of the AZ scaffold protein BRP revealed neuronal silencing decreased the protein's turnover. Although enlarged AZs are also observed in *rab3* mutants, activity reduction acted through an independent mechanism that required postsynaptic glutamate receptor-dependent signaling. Endogenous tagging of the Unc13B early AZ scaffold and the Unc13A late AZ scaffold revealed activity reduction decreased seeding of both early and late scaffolds, in contrast to *rab3* mutants. Together, these data indicate AZ maturation regulates presynaptic release mode and output strength, with neuronal activity shaping both AZ number and size across development.

## Significance Statement

How presynaptic development regulates neurotransmitter release output and the role of synaptic activity in active zone (AZ) maturation are unclear. Here, we perform birth dating and serial in vivo imaging of AZs over a multiday period during *Drosophila* larval development. We find synaptic maturation regulates the strength of synaptic output and the timing of spontaneous versus evoked release. Synaptic activity modulates AZ material accumulation and seeding of new release sites, with disruptions to neurotransmitter release reducing AZ number and driving enhanced AZ material accumulation at fewer release sites.

## Introduction

Synaptogenesis requires the formation of specialized points of contact between pre- and postsynaptic cells. Active zones (AZ) specialized for neurotransmitter release assemble at sites along the presynaptic axon that are opposed to postsynaptic neurotransmitter receptor clusters (Zhai and Bellen, 2004). AZs form an electron-dense cytomatrix containing multiple evolutionary conserved proteins, including cell adhesion molecules (neurexins, teneurins), scaffolding proteins [Liprin-α, Syd-1, RIM, RIM-Binding Protein (RBP), Unc13, BRP/ELKS/CAST], and Ca_v_2 voltage-gated Ca^2+^ channels (VGCCs; Zhen and Jin, 2004; Harris and Littleton, 2015; Ghelani and Sigrist, 2018; Chou et al., 2020; Emperador-Melero and Kaeser, 2020; Duhart and Mosca, 2022). A set of early scaffolding proteins initially assemble at release sites before the arrival of VGCCs and late scaffolds that cluster synaptic vesicles (SVs; Ghelani and Sigrist, 2018). Although most AZs contain a similar assortment of proteins, the distribution of these components is not uniform and can be rapidly modified (Weyhersmüller et al., 2011; Sheng et al., 2012; Sugie et al., 2015; Böhme et al., 2016; Akbergenova et al., 2018; Ghelani et al., 2023). How differential distribution of AZ components affects synaptic output is not clear. Similarly, mechanisms that shape AZ assembly and remodeling in response to changes in neuronal activity remain elusive.

*Drosophila* larval glutamatergic neuromuscular junctions (NMJs) have emerged as a useful model synapse to characterize AZ maturation and synaptic output (Jan and Jan, 1976; Broadie and Bate, 1993; Keshishian et al., 1996). NMJs form during late embryogenesis and expand throughout the subsequent 6 d of larval development (Harris and Littleton, 2015). Motoneurons (MNs) continue to add new synaptic boutons containing many AZs, with multiple pathways like bone morphogenetic protein and Wingless signaling coordinating NMJ growth (Chou et al., 2020). AZ maturation requires the accumulation of specific scaffolding proteins that can be resolved with live imaging through the cuticle using fluorescently tagged proteins of interest (Fouquet et al., 2009; Akbergenova et al., 2018). With the advent of quantal imaging that allows SV release to be measured at individual release sites at larval NMJs (Peled and Isacoff, 2011; Melom et al., 2013), how the specific molecular composition of AZs correlates with their spontaneous and evoked release probability (*P_r_*) can be measured. Indeed, prior quantal imaging at larval NMJs revealed a striking heterogeneity in AZ *P_r_*, ranging from silent synapses, spontaneous-only release sites, to those with evoked *P_r_* differing by ∼50-fold (Melom et al., 2013; Peled et al., 2014; Newman et al., 2017, 2022; Akbergenova et al., 2018; Sauvola et al., 2021; He et al., 2023; Jetti et al., 2023; Medeiros et al., 2024).

In the current study, we examine how AZ material accumulation regulates synaptic output and release mode and how reduced activity alters AZ maturation. By time-stamping synapses, we observe that presynaptic output dramatically increases as AZs mature over the course of several days. Although early scaffolding components such as Liprin-α, Syd1, and Unc13B are the first to arrive at developing AZs, their overall abundance correlates poorly with VGCC accumulation and the strength of evoked output at single release sites. In contrast, the abundance of late-arriving scaffolding components like RBP, BRP, and Unc13A, along with Cacophony (Cac, the *Drosophila* Ca_v_2 homolog), have a stronger contribution to synaptic efficacy. Maturing AZs that have not yet accumulated Cac channels appear as spontaneous-only sites that lack evoked release. In addition, the normal process of AZ formation and maturation is altered when neurotransmitter release is disrupted. Instead of seeding new AZs during development, silenced neurons form fewer and larger release sites and have reduced protein turnover of the AZ protein BRP. These data indicate neuronal activity regulates AZ seeding and material accumulation, with increases in functional output over time.

## Materials and Methods

### Drosophila stocks

*Drosophila melanogaster* were cultured on standard medium at 25°C. Male larvae were used for all experiments to facilitate crossing as fluorescently labeled Cac is located on the X chromosome. For EM experiments, larvae of both sexes were used. The Syt1^DN^ line used in this study was UAS-Syt1^C2BD356N,D362N^ (Guan et al., 2017). GluRIIB-GFP, GluRIIA-RFP, UAS-Liprin-α-mStrawberry, UAS-Syd1-mStrawberry, UAS-RBP-mCherry, and UAS-Rim-mCherry were provided by Stephan Sigrist (Freie Universität Berlin). The *rab3* null mutant (*rab3^rup^*) was provided by Ethan Graf (Amherst College). Other lines used include UAS-TeNT (BDSC #28837), sgRNA for *Cac* (BDSC #85863), RNAi for *Para* (BDSC #33923), *Rab3* RNAi (VDRC #330151), Ib-specific Gal4 GMR94G06 (BDSC #40701), endogenously CRISPR-tagged Cac-GFP (Gratz et al., 2019), and UAS-Cas9 (Guss et al., 2023). For AZ *P_r_* mapping, 44H10 (Mef2)-LexA (provided by Gerry Rubin, Janelia), LexAOp-myr-jGCaMP7s (Sauvola et al., 2021), and endogenously CRISPR-tagged Cac^TagRFP^ (provided by Kate O’Conner-Giles; Gratz et al., 2019) were used. A *GluRIIA* null mutant with a CRISRP-induced frame shift was provided by Dion Dickman (University of Southern California; Han et al., 2023).

### Generation of GluRIIE-mMaple, fluorescently labeled Unc13A/B, mEosEM BRP, and NEMOm-BRP transgenic strains

To generate GluRIIE-mMaple, GluRIIE sequence was obtained from plasmid FI04462 (DGRC stock 1621963), and mMaple was amplified from a plasmid (Addgene #141151). The two constructs were cloned into pBid-UASc (Addgene #35200) using EcoRI and XbaI.

To generate CRISPR-tagged Unc13A, four guide RNAs (gRNAs) were selected using the CRISPR Optimal Target Finder (Gratz et al., 2014): gRNA1 AGCTCGGCAACGATGGCATT; gRNA2 CATGCAGGTGTTACGCCAAA; gRNA3 AAAAAAAAAACGCTCTTGAG; and gRNA4 CCTGCCCTCAATTAAAGTAG. These gRNAs were fused with the pCFD5 expression vector (Addgene #73914) according to the Gibson assembly protocol using NEBuilder HighFidelity DNA Assembly Cloning Kit (E5520). mRuby was amplified from a plasmid (Addgene #105802) and flanked with right and left homology arms corresponding to the end of the first exon for Unc13A. Small flex regions were added before (ggcggaagc) and after (ggaggcagt) the mRuby sequence. A similar strategy was used to generate Unc13B tagged with mClover. Four gRNAs were assembled in pCFD5, targeting the area close to the end of the first exon corresponding to Unc13B: gRNA1 GTAGGCAATGAAACCGCTGT; gRNA2 AACCCTTCCGAACCGATTTA; gRNA3 AACCAGGGCTGTGAACCATT; and gRNA4 AAGATGAAATGATAAAGGAC. The mClover sequence was obtained from a plasmid (Addgene #105778) and flanked to right and left homology arms corresponding to an 800 base pair area close to the end of the first exon. These constructs were coinjected by BestGene into *vas*-Cas9 embryos (BDSC #51324) and positive transformants were selected by PCR amplification of sequences corresponding to mRuby and mClover.

To generate mEosEM-tagged BRP, we inserted mEosEM sequence (Paez-Segala et al., 2015) at genomic position 9530203 of the *brp* locus. This insertion site labels all BRP isoforms and was chosen based on previously successful insertions of GFP at this site (Mi{PT-GFSTF.0}brp[MI02987-GFSTF.0]; Nagarkar-Jaiswal et al., 2015).

gRNA sequences used were as follows:

TATCCATATCGCCCCAACCG
TGCACTTGCAATTGCGAGTT
GATGACCACGTCCCAGGAAC
Artificial gRNA sequence: GATGCCCGAAGGCTACGTGC
gRNA scaffold sequence:
GTTTTAGAGCTAGAAATAGCAAGTTAAAATaaggctagtccgttatcaacttgaaaaagtggcaccgagtcggtgc

All gRNA sequences and scaffolds were generated by PCR amplification from donor plasmid pCFD5. mEosEM sequence and homology regions were synthetized by Twist Bioscience. gRNAs and donor were combined in the pCFD5 vector according to the Gibson assembly protocol using NEBuilder HighFidelity DNA Assembly Cloning Kit (E5520). Artificial gRNA sequences with PAM region AGG were added to the area flanking 306 bp homology arms of *brp*, allowing linearization of donor DNA. The construct was injected by BestGene into nos-Cas9 embryos (BDSC #78782), and positive transformants were selected by detecting larval fluorescence, followed by PCR amplification and sequencing of genomic DNA in the insertion region.

To generate BRP with NEMOm at the N-terminus, multiple gRNA scaffolds were used based on previously reported sequences (Wang et al., 2024). Donor sequence containing NEMOm (Li et al., 2023) was flanked by *brp* homology arms and synthetized by Twist Bioscience. Homology arms were 500 base pairs upstream and downstream of the *brp* start codon. Both donor and gRNA sequences were combined by Gibson Assembly in a single pCFD5 vector. After injection of the construct into nos-Cas9 embryos (BDSC #78782) by BestGene, adults were crossed to a balancer line, and larvae with visible green fluorescent signal were preselected. Stocks produced from preselected larvae were PCR amplified and sequenced to confirm they matched the designed sequence. The following gRNA and gRNA scaffold sequences were used:

gRNA 1: CGCACACCAGAGCTAGTTAC
gRNA scaffold 1:
GTTTCAGAGCTATGCTGGAAACAGCATAGCAAGTTGAAATAAGGCTAGTCCGTTATCAACTTGAAAAAGTGGCACCGAGTCGGTGC
gRNA 2: GATCGACCGTCGGCAATTCC
gRNA scaffold 2:
GTTTGAGAGCAATGGTGGAAACACCATTGCAAGTTCAAATACGGCATGTCCGATATCAGCCTGAAAAGGCGGAAACGAGTCGTTTC
gRNA 3: GCGGCATGCAATCACACACG
gRNA scaffold 3:
GTTTGAGAGAGATGAGGGAAACCTCATCTCAAGTTCAAATAAGCGTACTGCGTTATCAAAGTGAAAACTTGGCCACGAGTCGTGGC
gRNA 4: GTTGCATTTAAGGTTGCATC
gRNA scaffold 4:
GTTGAAGAGTGATGTGGGAAACCACATCACAAGTTTCAATAAGGGTACTCCGTTATCAATGTGAAAACATGGCGACGAGTCGTCGC
gRNA 5: GTGGAACCGGAGGTTGCAGT
gRNA scaffold 5:
GTTACAGACCAATGCTGACAACAGCATTGGAAGTTGTAATAAGCCTAGTGCGTTATCAAGTTGAAAAACTGCCCACGAGTCGTGGG
Artificial gRNA: GACCGGTGAAGGCTACGTGC
Artificial scaffold gRNA:
GTTTTAGAGCTAGAAATAGCAAGTTAAAATAAGGCTAGTCCGTTATCAACTTGAAAAAGTGGCACCGAGTCGGTGC

The artificial gRNA sequence GACCGGTGAAGGCTACGTGC with a PAM site (AGG) was added to the flanking regions of the donor homology arms to provide linearization of the donor.

### Confocal imaging and data analysis

Confocal images were acquired using a PerkinElmer system equipped with a spinning-disk confocal head (CSU-X1; Yokogawa) and Hamamatsu C9100–13 ImagEM EM CCD camera (Hamamatsu). A Zeiss plan-APOCHROMAT 63× objective with 1.40 NA was used for imaging of stained preparations. A 3D image stack was acquired for each NMJ imaged. Image analysis was performed in Volocity 3D Image Analysis software (Quorum Technologies) and 3D stack images were merged into a single plane for 2D analysis using the max intensity projection algorithm from Volocity 6.5.0 software. Analysis of Cac, BRP, RBP, GluRIIB, and GluRIIA intensities were performed via the “find object” algorithm that detects fluorescent areas above a preset threshold. All images in the same dataset were tiled together, and one algorithm of analysis was applied to each image. Immunoreactive proteins were imaged at segments A3 and A4 of Muscle 4 (M4) for all experiments unless indicated. For M1-specific manipulations, neighboring M9 NMJs in the same larvae were used as control.

### Immunocytochemistry

Larvae were dissected in HL3 solution and fixed in 4% paraformaldehyde for 10 min, washed in Ca^2+-^free HL3, and blocked and permeabilized for 1 h in PBS containing 0.1% Triton X-100, 2.5% NGS, 2.5% BSA, and 0.1% sodium azide. Samples were incubated overnight at 4°C in blocking solution containing primary antibodies and then washed for 1 h in blocking solution. Samples were incubated for 1 h at room temperature in blocking solution containing fluorophore-conjugated secondary antibodies. Primary antibodies used in this study were mouse anti-BRP at 1:500 (nc82 DSHB) and rabbit anti-RBP at 1:500 (provided by Stephan Sigrist). Secondary antibodies used in this study were goat anti-mouse Alexa Fluor 607-, 546-, or 488-conjugated anti-mouse IgG at 1:1,000 (Invitrogen, #A21237, #A11030, and #A32723) and goat anti-rabbit Alexa Fluor 647-conjugated IgG at 1:1,000 (Molecular Probes #4414). For HRP staining, samples were incubated in DyLight 649-conjugated HRP at 1:500 (#123-605-021; Jackson ImmunoResearch Laboratories). Samples were mounted in VECTASHIELD (Vector Laboratories) before imaging.

### Optical AZ *P_r_* mapping

Third instar larvae expressing lexOP-myr-jGCaMP7s with the postsynaptic mef2-LexA (44H10) driver were dissected in Ca^2+^-free HL3 containing 20 mM MgCl. After dissection, preparations were maintained in HL3 with 20 mM MgCl and 1.0 mM Ca^2+^ for 5 min. Optical AZ *P_r_* mapping was performed on a PerkinElmer system equipped with a spinning-disk confocal head (CSU-X1; Yokogawa) and Hamamatsu C9100–13 ImagEM EM CCD camera (Hamamatsu) as previously described (Akbergenova et al., 2018). An Olympus LUMFL N 60× objective with a 1.10 NA was used to acquire GCaMP7s imaging data at 8 Hz. A dual channel multiplane Z-stack at M4 NMJs in segments A3 and A4 was acquired at the beginning of each experiment to identify AZ position using expression of Cac^TagRFP^ or fluorescently labeled AZ scaffold proteins (Liprin-α, Syd1, RBP, or Unc13A). Single focal plane videos were then recorded, while MNs were stimulated with a suction electrode at 1 Hz for 3–5 min. The red channel was recorded every 20 s to make sure AZ position did not change. Slight *z*-drift was manually corrected during each imaging session, while imaging sessions with significant muscle movement were discarded. For experiments using time-stamped postsynaptic densities (PSDs), UAS-GluRIIE-mMaple was expressed postsynaptically using Mef2-Gal4 (BDSC #27390). AZ or PSD position was reimaged every 25 s during constant video recording of myrGCaMP7s. The dual channel stack was merged into a single plane using the max intensity projection algorithm from Volocity 6.5.0 software (Quorum Technologies). Images of all AZs or PSDs were added to the myrGCaMP7s stimulation video and detected automatically using the spot finding function of Volocity. Equal size ROIs were assigned to each. In cases where the software failed to label visible AZs or PSDs, ROIs were added manually. GCaMP7s peak flashes were detected and assigned to ROIs based on centroid proximity with a 0.8 µm threshold. Evoked events were identified as frames with three or more simultaneous GCaMP events recorded at the NMJ. The time and location of events were imported into Excel for further analysis. Evoked GCaMP events per ROI were divided by the number of stimulations to calculate AZ *P_r_*.

### GluRIIE-mMaple and mEosEM BRP photoconversion

GluRIIE-mMaple larvae were collected after growing at 25°C, briefly washed in HL3 saline, and placed ventral side up on a glass slide with a drop of halocarbon oil. To restrict larval movement, a cover glass was placed on top of larvae and attached with plastalina clay. Photoconversion (PC) was performed using a 10× Zeiss Plan-APOCHROMAT objective. Samples were illuminated using a Lumen Dynamics X-Cite XYLIS LED light source with a 360/51 BP filter for 15 s. To photoconvert mEosEM BRP, we collected early third instar larvae, briefly washed them in HL3 saline, and anesthetized them via isoflurane exposure for 40 s. Anesthetized larvae were placed on a glass slide with a drop of halocarbon oil. Larvae were positioned laterally to expose M1 and M9. To restrict larval movement, a cover glass was placed on top of larvae and attached with plastalina clay. PC was performed using a Dragonfly Confocal Microscope System (Oxford Instruments). M1 and M9 NMJs were identified under illumination with a 488 laser with minimal laser power to prevent bleaching. Only NMJs innervating segment A2 or A3 were photoconverted. A *Z*-stack through the NMJs was photoconverted using a 405 nm laser for 30 s. The same area was imaged again using a 488 nm laser to verify no residual green fluorescence remained. Photoconverted larvae were either dissected and imaged immediately or allowed to recover for 24 h at 25°C and then dissected, fixed and imaged. A ∼30% lethality rate was observed after isoflurane anesthesia, and only larvae that recovered within 15 min were included in the analysis.

### Ca^2+^ influx detection using BRP-NEMOm

Third instar larvae with endogenous NEMOm-BRP and cac-TagRFP and expressing UAS-TeNT with the MN1-Ib–specific Gal4 driver were dissected in cold Ca^2+^-free HL3 solution. To prevent damage to M1 during dissection, we cut the larvae on a lateral side and performed the imaging on single A2 and A3 hemisegments. Prior to imaging, HL3 containing 1.5 mM Ca^2+^ was added. Nerve bundles to the A2 or A3 segment were positioned into a suction electrode, and samples were stimulated at 10 Hz. *Z*-stacks from M1 and M9 were imaged.

### Electron microscopy

Third instar larvae were dissected in Ca^2+-^free HL3.1 solution and fixed in 1% glutaraldehyde, 4% formaldehyde, and 0.1 M sodium cacodylate for 10 min at room temperature. To identify specific muscle NMJs, we positioned the cactus pins next to M1 and transferred the larvae into the glass vials. Fresh fixative was added, and samples were incubated for 1 h at room temperature. After washing samples in 0.1 M sodium cacodylate and 0.1 M sucrose, samples were stained for 30 min in 1% osmium tetroxide and 1.5% potassium ferrocyanide in 0.1 M sodium cacodylate solution. After washing with 0.1 M sodium cacodylate, samples were stained for 30 min in 2% uranyl acetate and dehydrated through a graded series of ethanol and acetone, before embedding in epoxy resin (Embed 812; Electron Microscopy Sciences). Thin sections (50–60 nm) were collected on Formvar/carbon-coated copper slot grids and contrasted with lead citrate and uranyl acetate. Sections were imaged at 49,000× magnification at 80 kV with a Tecnai G2 electron microscope (FEI) equipped with a charge-coupled device camera (Advanced Microscopy Techniques). Type Ib boutons at M1 were analyzed. For SV counting, T-bars at Ib boutons were identified, and the Volocity software was used to measure T-bar and PSD length. All data analyses were done blinded.

### MINFLUX data acquisition

MINFLUX data were collected on a commercial Abberior Instruments MINFLUX setup (Abberior Instruments) as previously described (Schmidt et al., 2021). The system was equipped with a 100×/1.4 NA magnification oil immersion lens, a 640 nm continuous-wave laser for exciting the CAGE635-labeled BRP structures in both confocal and MINFLUX mode, a 488 nm pulsed laser for exciting the AF488-colabeled BRP structures in a confocal mode, and a 405 nm continuous-wave laser for photoactivating single CAGE635 molecules. MINFLUX imaging was performed with standard 2D/3D imaging sequences provided by Abberior Instruments and a 640 nm excitation power of 120 μW (2D)/250 μW (3D) in the first iteration, increasing over the subsequent iterations to reach 720 μW (2D)/1.5 mW (3D) in the final one, as measured in front of the microscope body. The emission of individual CAGE635 molecules was detected between 650 and 720 nm with the pinhole set to 0.83 AU. Before photoactivating CAGE635 during the MINFLUX measurements, confocal images of NMJs were acquired using the AF488 signal of BRP to serve as a guide and identify regions of interest. The sample position was actively stabilized on the backscattered light of gold beads illuminated with 975 nm in a widefield mode. The Abberior Instruments Inspector software with MINFLUX drivers was used to operate the system. MINFLUX data were rendered in a pixel-based fashion.

### Statistical analysis

Statistical analysis and graphing were performed with GraphPad Prism. Statistical significance was determined using Student's *t* test for comparisons between two groups or a one-way ANOVA followed by Tukey's multiple-comparison test for three or more groups unless noted. In the figures, the center of each distribution is plotted as the mean value and reported in the figure legends as the mean ± SEM with the corresponding number of samples (*n*). In all cases, *n* represents the number of individual NMJs analyzed unless noted. The number of larvae used per group in each experiment is indicated in the text. Asterisks in the figures denote the following *p* values: *p ≤ 0.05; **p ≤ 0.01; ***p ≤ 0.001; and ****p ≤ 0.0001. Raw values and statistical details for experiments are provided in Data S1.

## Results

### A genetic toolkit for time-stamping synapse age

Prior quantal imaging studies at *Drosophila* NMJs revealed a ∼50-fold heterogeneity in evoked *P_r_* across the hundreds of AZs formed by a single MN (Peled and Isacoff, 2011; Melom et al., 2013; Newman et al., 2017, 2022; Akbergenova et al., 2018; Jetti et al., 2023; Medeiros et al., 2024). One model for this heterogeneity predicts each AZ is fated to have a distinct output strength that is maintained over the life of that individual AZ. This could arise from AZ components being delivered in variable abundance and/or composition that are deposited together at forming synapses or through the presence of unique components that direct future output to a set *P_r_*. An alternative model is that AZs are initially assembled with poor release capacity and mature over development to a higher *P_r_* state, with heterogeneity reflecting distinct stages of age-dependent maturation across the AZ population. Serial imaging of NMJ growth in briefly anesthetized *Drosophila* larvae provided support for the second model, suggesting newly formed AZs are weaker than their more mature counterparts (Akbergenova et al., 2018). However, the rapid rate of AZ addition during larval growth makes it difficult to follow defined AZs across multiple imaging sessions.

**Figure 1.**
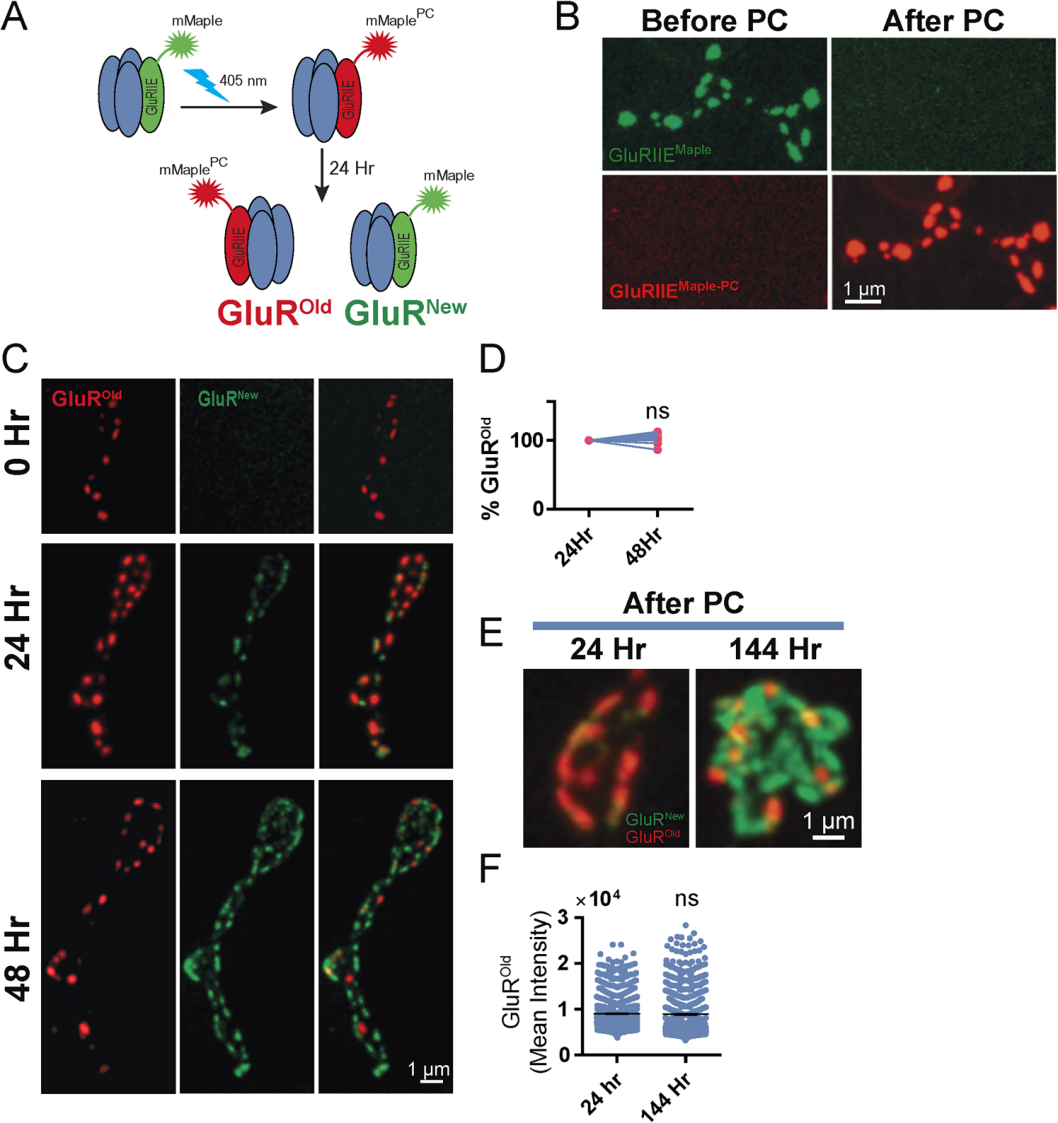
GluRIIE-mMaple PC to timestamp synapse age. ***A***, Schematic showing PC of GluRIIE^Maple^ from green to red. All existing PSDs at the time of PC contain red^+^ GluRIIE (PSD^Old^), while newer PSDs formed after PC contain green^+^/red^−^ GluRIIE (PSD^New^). ***B***, Complete PC of larval NMJ PSDs containing GluRIIE^Maple^ from green^+^ to red^+^ after 15 s transcuticular exposure to 405 nm light. ***C***, Serial imaging of the same M4 NMJ following brief anesthetization at three time points (0, 24, 48 h). PC was performed at timepoint 0 in the first instar stage. Although new PSDs incorporated nonsynaptic red^+^ GluRIIE during the first day post-PC (middle panel), only non-PC green^+^/red^−^ GluRIIE was present at PSDs that formed after 24 h (bottom panel). ***D***, The percentage of red^+^ GluRIIE PSDs remains unchanged between 24 and 48 h PC, indicating new PSDs formed after 24 h contain only newly synthesized GluR^New^ (24 h, 100%; *n* = 15 NMJs in 5 larvae; 48 h, 101.4% ± 0.85; *n* = 15 in 5 larvae; *p* = 0.11). ***E***, Representative single bouton images of PSDs containing GluR^New^ and GluR^Old^ at 24 and 144 h post-PC. ***F***, Red^+^ GluR^Old^ fluorescent intensity at larval NMJ PSDs is stable and does not undergo significant decay between 24 and 144 h post-PC (Day 1, 9,070 ± 97.3 average RFU; *n* = 1,198 PSDs in 4 larvae; Day 6, 8,910 ± 162.2; *n* = 858 PSDs in 4 larvae; *p* = 0.3725). Statistical significance determined with Student's *t* test, ns, not significant. Raw values and statistical details are provided in Data S1.

To more precisely examine how synapse age contributes to presynaptic output at individual AZs, we developed a new toolkit for time-stamping synapses. mMaple is a genetically encoded fluorophore that undergoes complete green-to-red PC upon illumination with 405 nm light (McEvoy et al., 2012). By attaching mMaple to the core postsynaptic glutamate receptor subunit GluRIIE in transgenic animals, all PSDs opposed to presynaptic AZs at the NMJ can be reliably labeled (Fig. 1*A*). Brief 15 s illumination at 405 nm through the cuticle of intact first instar larvae was sufficient to generate complete transition of the entire pool of GluRIIE^Maple^ from green to red at M4 NMJs (Fig. 1*B*). Serial imaging of NMJs identified a small pool of nonsynaptic red^+^ GluRIIE^Maple^ that continued to incorporate into PSDs during the first day after PC, resulting in a mild increase in the number of red^+^ GluRIIE-positive PSDs (Fig. 1*C*). However, 48 h post PC, all newly formed PSDs contained only non-PC green^+^ GluRIIE^Maple^ (hereafter referred to as GluR^New^; Fig. 1*C*,*D*). Quantification of the number of PC red^+^ GluRIIE^Maple^-positive PSDs (hereafter referred to as GluR^Old^) revealed no change between 24 and 48 h (Fig. 1*D*), indicating the entire pool of previously PC GluRIIE^Maple^ had inserted into PSDs within a day and did not undergo lateral movement into newly formed synapses. Although no new GluR^Old^ PSDs were observed, NMJs continued to grow with the number of GluR^New^ PSDs increasing as expected (Fig. 1*C*,*E*).

To determine the extent of GluR turnover, average fluorescent intensity of red^+^ GluR^Old^ PSDs was compared at Day 1 and Day 6 post-PC. Any decay in fluorescence of GluR^Old^ likely reflects turnover, as red-shifted mMaple is highly stable and cannot spontaneously transition back to the green state. No significant decrease in GluR^Old^ fluorescence was observed over this 5 d interval (Fig. 1*E*,*F*; Data S1). These data indicate GluR clusters at *Drosophila* NMJs are highly stable and do not undergo robust turnover during larval development, consistent with prior imaging of GluRs (Rasse et al., 2005). The absence of GluR^Old^ at newly formed PSDs later in development also indicates GluRs do not undergo lateral movement between PSDs, similar to observations using mMaple-tagged Cac (the sole *Drosophila* Ca_v_2 Ca^2+^ channel) that is retained within individual presynaptic AZs (Cunningham et al., 2022). Together, these data establish GluRIIE^Maple^ as a tool for time-stamping synapses.

### Synaptic output and release mode are dependent on AZ age and maturation state

**Figure 2.**
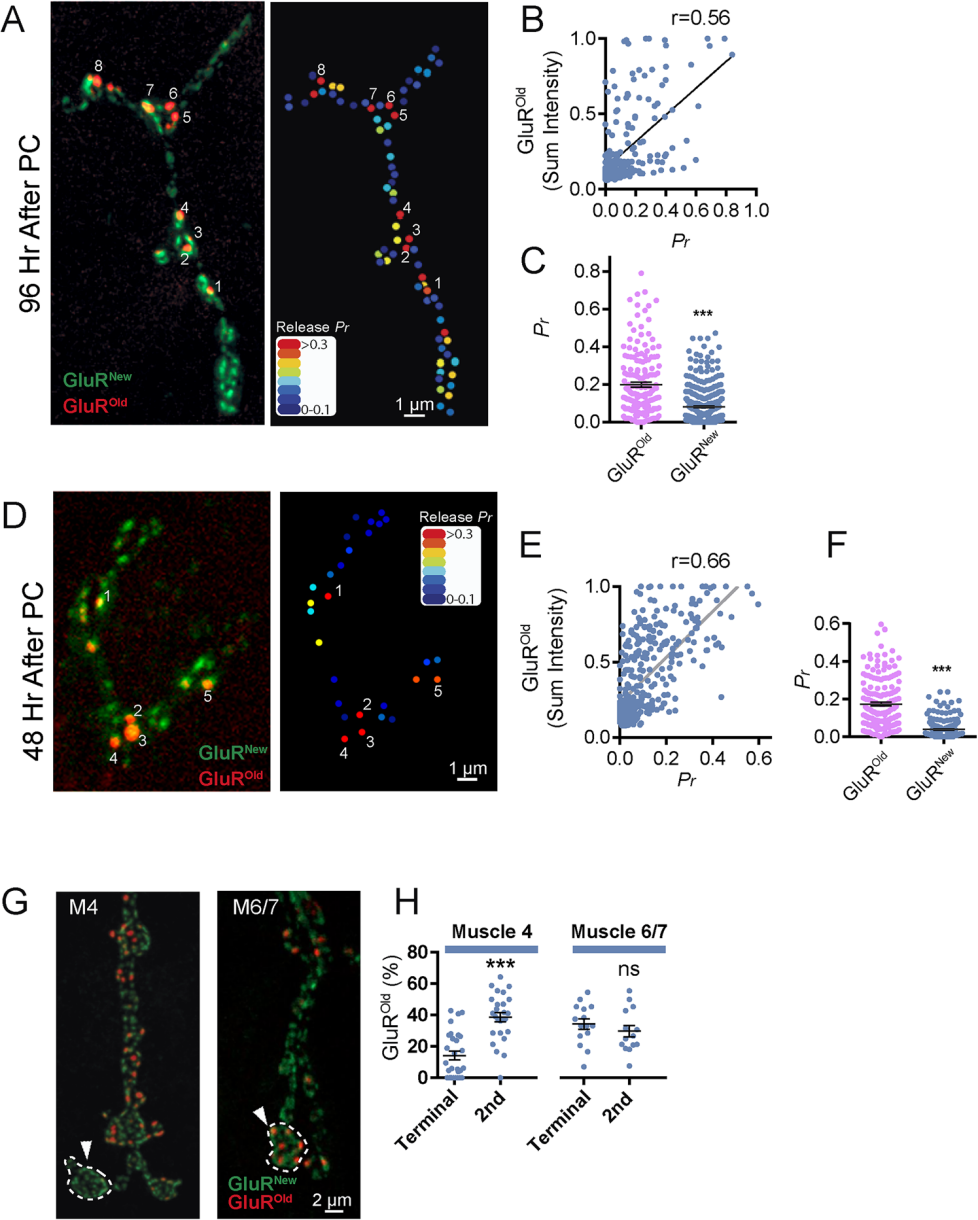
Enhanced evoked synaptic output at older PSDs. ***A***, Representative larval M4 NMJ imaged 96 h after PC with GluR^Old^ PSDs individually numbered (left panel). The corresponding evoked *P_r_* heatmap following quantal imaging with myrGCaMP7s is shown on the right, revealing AZs opposed to the oldest PSDs are among the strongest release sites. ***B***, Correlation between PSD age, determined by GluR^Old^ sum fluorescent intensity of individual PSDs, and evoked *P_r_* (Pearson's *r* value = 0.56). GluR^Old^ sum intensity was normalized for each NMJ across animals, with the max sum fluorescence PSD signal set to 1.0 for each corresponding NMJ. ***C***, Average evoked *P_r_* for AZs opposed to older PSDs containing any red^+^ GluR^Old^ compared with younger PSDs with only green^+^/red^−^ GluR^New^ (red^+^ PSDs, 0.193 ± 0.013; *n* = 174 PSDs from 5 larvae; red^−^ PSDs, 0.081 ± 0.005; *n* = 378 PSDs from 5 larvae; *p* < 0.0001). ***D***, Representative larval M4 NMJ imaged 48 h after PC with GluR^Old^ PSDs individually numbered (left panel). The corresponding evoked *P_r_* heatmap following quantal imaging with myrGCaMP7s is shown on the right. ***E***, Correlation between PSD age determined by red^+^ GluR^Old^ fluorescent intensity and evoked *P_r_* (Pearson's *r* value = 0.66). ***F***, Average evoked *P_r_* for AZs opposed to older PSDs containing any red^+^ GluR^Old^ compared with younger PSDs with only green^+^/red^−^ GluR^New^ (red^+^ PSDs, 0.174 ± 0.01; *n* = 179 PSDs from 5 larvae; red^−^ PSDs, 0.0397 ± 0.004; *n* = 192 PSDs from 5 larvae; *p* < 0.0001). ***G***, Representative images of NMJ growth at M4 (left) and M6/7 (right) 4 d after PC of GluRIIE^Maple^ first instar larvae. Note the addition of terminal boutons lacking older red^+^ PSDs (arrow) at M4 versus the internal addition of new boutons lacking red^+^ PSDs at M6 (white outline and arrows). ***H***, Quantification of the percent of GluR^Old^ PSDs to all PSDs at the terminal bouton versus the second bouton from the end. Older PSDs are enriched in the terminal bouton at M6/7 NMJs (terminal bouton GluR^Old^ positive PSDs as a percent of all PSDs, M6/7, 34.3% ± 3.4; *n* = 15 NMJs from 5 larvae; M4, 14.2% ± 2.7; *n* = 29 NMJs from 5 larvae; second bouton from end, M6/7, 29.7% ± 3.7; *n* = 14 NMJs from 5 larvae; M4, 38.6% ± 3.0; *n* = 26 NMJs from 5 larvae). Statistical significance determined with Student's *t* test, ns, not significant. Asterisks denote the following *p* value: ***p ≤ 0.001. Raw values and statistical details are provided in Data S1.

To examine the effect of age-dependent synapse maturation on presynaptic output, we performed quantal imaging of single SV release events at individual AZs using postsynaptically expressed membrane-tethered myrGCaMP7s (Movie 1) as previously described (Akbergenova et al., 2018; Sauvola et al., 2021; Jetti et al., 2023). Animals expressing myrGCaMP7s and GluRIIE^Maple^ were PC during the early second instar larval stage, and quantal imaging was performed 4 d later to generate AZ *P_r_* maps. The myrGCaMP7s signal is much dimmer than GluRIIE^Maple^ at rest and does not interfere with identification of GluR^New^ PSDs. Similarly, the dramatic increase in myrGCaMP7s fluorescence following SV fusion and subsequent postsynaptic opening of GluRs allows simultaneous imaging of synaptic activity on top of baseline GluRIIE^Maple^. Older PSDs had more GluR^Old^ and brighter red fluorescence compared with newer PSDs that accumulated only GluR^New^ (Fig. 2*A*). Total GluR^Old^ intensity at individual PSDs was highly correlated with *P_r_* (Pearson's *r* value = 0.56; Fig. 2*A*,*B*), indicating evoked output of the corresponding AZ was strongly dependent on synapse age. Comparison of all sites that contained any amount of GluR^Old^ with newer synapses that contained only GluR^New^ revealed a ∼2.5-fold difference in *P_r_* (Fig. 2*C*). These data indicate older AZs establish and maintain higher evoked *P_r_* across larval development.

**Movie 1.** Quantal imaging of a M4 NMJ using 1 Hz nerve stimulation in a third instar larva expressing LexAOp-myr-GCaMP7s postsynaptically with 44H10 (Mef2)-LexA. Evoked release from individual AZs is observed as synchronous increases in fluorescence across distinct populations of PSDs. Individual spontaneous mini events can be observed between stimulation. [View online]

Quantal imaging after 4 d of PC does not allow precise determination of the output of newer release sites, as GluR^New^ PSDs are indistinguishable whether they formed during Day 1 or Day 4 after PC. To more precisely examine AZ age and release strength, we performed quantal imaging over a shorter 2 d interval after PC of early second instar larvae (Fig. 2*D*). A stronger correlation was observed for evoked *P_r_* at sites containing GluR^Old^ versus newly formed PSDs that were <1 d old and contained only GluR^New^ (Pearson's *r* value = 0.66; Fig. 2*E*). Indeed, a more than fourfold difference in *P_r_* between older synapses containing any GluR^Old^ signal was observed compared with those without any (Fig. 2*F*). Together, these data indicate synapse age and the strength of evoked release at individual AZs are interlinked. Given these variables are not perfectly correlated, other factors are also likely to contribute to presynaptic strength.

Prior studies identified heterogeneity in synaptic transmission strength along some *Drosophila* larval motor axons, with terminal boutons showing enhanced Ca^2+^ influx and release (Guerrero et al., 2005). This graded transmission was not uniform across all MNs, as M6/7 MNs displayed enhanced output at terminal boutons while M4 MNs lacked this effect (Lnenicka et al., 2006). Molecular mechanisms explaining enhanced release from terminal boutons and differences across MN types remain unclear. Given NMJ expansion can result in individual release sites changing their relative position during larval development, we hypothesized distinct patterns of bouton addition during synaptic growth might contribute to the observed release heterogeneity. To compare patterns of NMJ growth at M6/7 versus M4, the relative abundance of GluR^Old^ PSDs was quantified at individual boutons from proximal to distal after several days of growth post-PC (Fig. 2*G*,*H*). Axon terminals of M6/7 MNs commonly grew by “stretching” and adding new internal boutons such that terminal boutons contained more GluR^Old^ PSDs. In contrast, M4 MNs often grew by budding new branches from the end of the axon, resulting in terminal boutons lacking or having fewer older PSDs. These distinct patterns of synaptic growth and AZ addition, along with the 2.5-fold enhancement of mature AZs at terminal boutons of M6/7 NMJs, provide an underlying mechanism to explain differences in graded transmission between these two MN subtypes.

**Figure 3.**
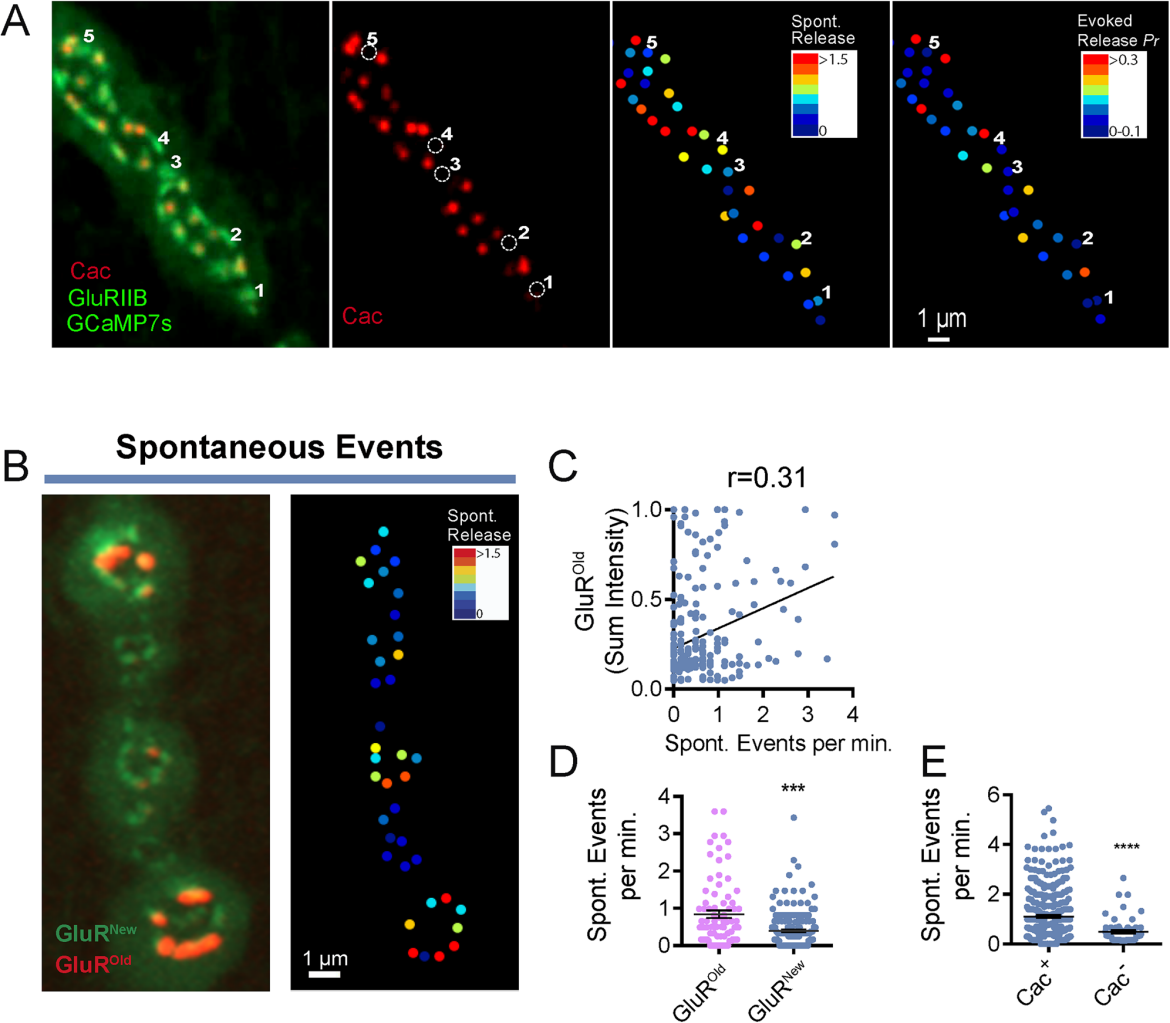
AZs lacking Cac can support spontaneous fusion. ***A***, Representative third instar M4 NMJ from a larva expressing GluRIIB-GFP and Cac^RFP^. Several PSDs lacking opposed Cac^RFP+^ signal are individually numbered. Cac^RFP^ signal is shown in Panel 2, with spontaneous activity (release events per minute) presented as a heat map at AZs opposed to GluRIIB^+^ PSDs in Panel 3. The corresponding evoked *P_r_* heatmap is shown on the right, highlighting lack of evoked release at Cac^−^ AZs. AZs lacking Cac can display low (Sites 1 and 5), moderate (Site 3), or high (Sites 2 and 4) rates of spontaneous release with no corresponding evoked output. ***B***, Representative third instar larval M4 NMJ imaged 96 h after PC to identify GluR^Old^ PSDs (left panel). The corresponding heatmap for spontaneous activity (release events per minute) is shown on the right. ***C***, Correlation between PSD age determined by red^+^ GluR^Old^ fluorescent intensity and spontaneous release rate (Pearson's *r* value = 0.31). ***D***, Spontaneous release rate for AZs opposed to older PSDs containing any red^+^ GluR compared with younger PSDs with only green^+^/red^−^ GluR (red^+^ synapses, 0.841 ± 0.1 events per min; *n* = 80 AZs from 4 larvae; red^−^ synapses, 0.398 ± 0.04; *n* = 157 AZs from 4 larvae; *p* < 0.0001). ***E***, Spontaneous release rate for AZs containing Cac^RFP^ compared with those without (Cac^RFP+^, 1.11 ± 0.05 events per min; *n* = 366 AZs from 5 larvae; Cac^RFP−^, 0.497 ± 0.07; *n* = 55 AZs from 5 larvae; *p* < 0.0001). Statistical significance determined with Student's *t* test; ns, not significant. Asterisks denote the following *p* values: ***p ≤ 0.001 and ****p ≤ 0.0001. Raw values and statistical details are provided in Data S1.

In addition to evoked release, single SVs also fuse through an action potential–independent mechanism to generate spontaneous “mini” events. We previously determined that ∼10% of the AZ population at mature third instar larval NMJs display only spontaneous release and lack evoked fusion (Akbergenova et al., 2018). Given the role of maturation for evoked *P_r_*, spontaneous-only AZs might reflect an early developmental stage prior to accumulation of Cac channels required to support evoked release. To determine if spontaneous fusion could occur at AZs lacking Cac, quantification of evoked *P_r_* and spontaneous release rate was performed in larvae expressing endogenously CRISPR-tagged Cac^TagRFP^ (Gratz et al., 2019). Comparison of heatmaps for the spontaneous release rate and evoked *P_r_* revealed a population of AZs capable of spontaneous fusion but lacking evoked release and detectable Cac (Fig. 3*A*). Although these data suggest spontaneous-only sites represent an immature state of AZ development, it is unclear if spontaneous release rate is enhanced during AZ maturation as observed for evoked *P_r_*. To correlate synapse age and spontaneous release efficacy, we performed quantal imaging 4 d following PC in GluRIIE^Maple^ larvae. Although a positive correlation between spontaneous release rate and PSD age was observed (Pearson's *r* value = 0.31; Fig. 3*B*,*C*), the correlation was significantly less than for evoked *P_r_* (*r* = 0.56; Fig. 2*B*). Overall, AZs opposed to sites that contained any GluR^Old^ had a ∼2-fold increase in spontaneous release rate compared with sites containing only GluR^New^ (Fig. 3*D*). Similarly, quantifying spontaneous release rate in larvae expressing endogenously CRISPR-tagged Cac^RFP^ revealed that Cac^RFP+^ AZs displayed a 2.2-fold increase in spontaneous release rate over AZs lacking Cac (Fig. 3*E*). We conclude that spontaneous-only AZs largely represent an early developmental state and that evoked and to a lesser extent spontaneous release become more efficient as AZs accumulate scaffolding proteins and VGCCs during maturation.

### Sequential addition and accumulation of AZ proteins at nascent synapses

**Figure 4.**
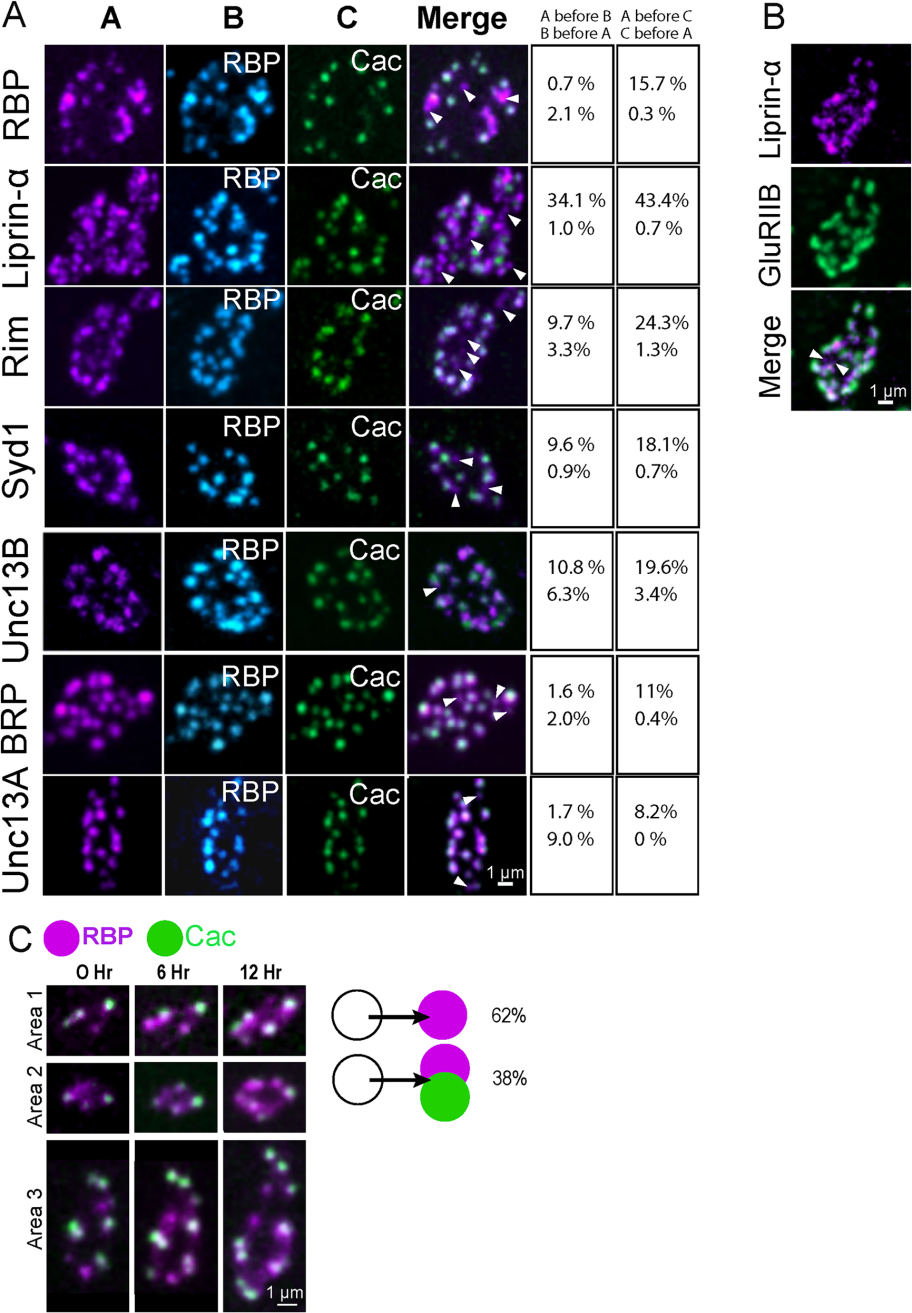
Time course for protein accumulation during AZ maturation. ***A***, Representative second instar M4 synaptic boutons with an analysis of the distribution of fluorescent puncta for the indicated AZ proteins (left panel ***A***) in relation to immunostained RBP (middle panel ***B***) and endogenous CRISPR GFP-tagged or RFP-tagged Cac (right panel ***C***). The merged image shows labeling for Cac and the AZ protein indicated in panel ***A***, with representative AZs lacking Cac indicated by white arrows. The right panels show the percentage of individual puncta for each AZ protein lacking RBP or Cac (top values) versus RBP or Cac puncta lacking the indicated AZ protein (bottom values). *N* = 7 NMJs from two larvae for each correlation. ***B***, Representative image of a second instar M4 synaptic bouton showing Liprin-α puncta compared with the distribution of GluRIIB. Most puncta overlap, although a few are positive for Liprin-α only (white arrows). ***C***, Serial imaging of RBP and Cac appearance at second instar M4 AZs over three time points indicate RBP accumulates before Cac arrival. The appearance of new RBP or Cac accumulations within 6 h intervals was scored (*n* = 10 NMJs from 3 larvae), revealing 62% of newly formed puncta are only RBP^+^ and 38% are RBP^+^ and Cac^+^. No case of Cac at newly formed AZs lacking RBP was observed. Raw values and statistical details are provided in Data S1.

Although a number of transport mechanisms are implicated in material delivery to synapses (Goldstein et al., 2008; Rizalar et al., 2021; Petzoldt, 2023), it is unclear if most AZ components are cotrafficked and delivered as preassembled modules. At *Drosophila* NMJs, a sequential process of AZ assembly has been observed, with Liprin-α and Syd1 arriving early during AZ formation (Owald and Sigrist, 2009). To quantify the pattern of accumulation for a broad range of AZ proteins, we analyzed fluorescently tagged lines expressing Liprin-α, Syd1, BRP, RIM, RBP, Cac, Unc13B, and Unc13A at early second instar larval NMJs when AZ addition is robust. The presence of these proteins across the AZ population was correlated with the distribution of endogenously CRISPR-tagged Cac channels (Cac^GFP^ or Cac^RFP^) and endogenous RBP, a late AZ scaffold, detected by antibody staining (Fig. 4*A*). Liprin-α was the earliest arriving AZ protein detected using this colocalization assay, with 34.1% of Liprin-α^+^ puncta lacking RBP and 43.4% lacking Cac. To determine if Liprin-α^+^ puncta were newly formed AZs or accumulations of the protein outside of release sites, we quantified the presence of co-opposed postsynaptic GluRIIB^+^ receptor fields. Of the Liprin-α puncta, 93.3% were opposed to GluRIIB^+^ PSDs (Fig. 4*B*), indicating most Liprin-α^+^ clusters detected using this method represent AZs in various states of maturation. RIM and Syd1 also appeared early at forming AZs, with 9.7% of RIM^+^ AZs lacking RBP and 24.3% lacking Cac, while 9.6% of Syd1^+^ AZs lacked RBP and 18.1% lacked Cac (Fig. 4*A*). In contrast, BRP arrived later, with only 1.6% of BRP^+^ AZs lacking RBP and 11% lacking Cac, similar to prior observations (Fouquet et al., 2009). To assay the arrival and distribution of the two Unc13 spicing isoforms (Böhme et al., 2016), CRISPR was used to endogenously tag Unc13B with mClover and Unc13A with mRuby. Unc13B was an early arriving AZ protein as previously observed (Böhme et al., 2016), with 10.8% of Unc13B^+^ AZs lacking RBP and 19.6% lacking Cac. Unc13A followed a similar pattern to the later-arriving scaffolds BRP and RBP, with only 1.7% of Unc13A^+^ AZs lacking RBP and 9% lacking Cac (Fig. 4*A*).

Although differential accumulation of proteins observed in fixed imaging suggests a sequential AZ maturation process, it doesn't provide a time course for AZ assembly. To examine AZ maturation over time, serial imaging of briefly anesthetized larvae expressing fluorescently tagged RBP and Cac was performed at 0, 6, and 12 h time points (Fig. 4*C*). The appearance of new RBP^+^ AZ clusters and their subsequent accumulation of Cac was scored across the three imaging sessions. All RBP^+^ AZs lacking Cac at timepoint 0 had accumulated Ca^2+^ channels by the 12 h timepoint. As such, Cac must arrive within an interval shorter than 12 h. We next focused on comparing Cac accumulation at RBP^+^ AZs within a shorter 6 h interval. If a RBP^+^ AZ was already present, Cac was observed in 100% of cases after 6 h. For 62% of cases where new RBP clusters appeared during a 6 h imaging window, Cac was not detected at these AZs. Newly formed RBP^+^ clusters that also contained Cac appeared 38% of the time, suggesting ∼4 h is required for Cac channels to arrive after RBP accumulation. Consistent with other assays measuring AZ protein accumulation at *Drosophila* NMJs (Rasse et al., 2005; Graf et al., 2009; Owald et al., 2012; Özel et al., 2019; Petzoldt et al., 2020), our data support a sequential addition process where Liprin-α arrives first, followed by a cohort of early scaffolds (Syd1, RIM, and Unc13B). BRP, RBP and Unc13A arrive at AZs later and around the same time, with the VGCC Cac following several hours after late scaffolds accumulate.

### Early AZ scaffold levels correlate poorly with evoked release, while late AZ scaffold abundance predicts presynaptic release output

**Figure 5.**
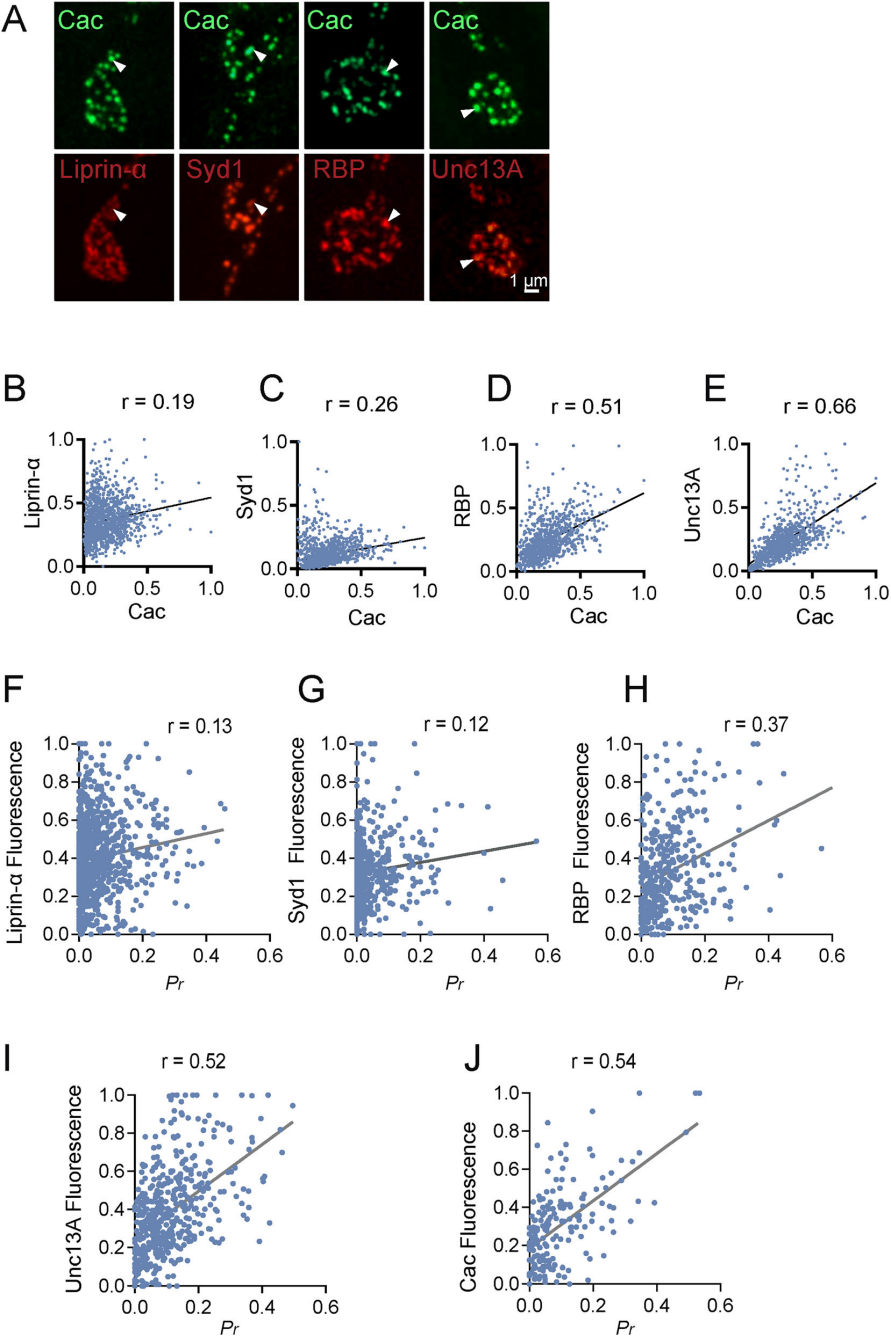
The abundance of early AZ scaffolds poorly predicts Cac accumulation and *P_r_*. ***A***, Representative images of third instar M4 synaptic boutons expressing endogenous CRISPR-tagged Cac^GFP^ (top row) and the indicated AZ scaffolding protein (bottom row). One of the brightest Cac^+^ puncta in each bouton is highlighted (white arrows) to compare abundance with the indicated scaffolding protein. ***B–E***, Correlation between AZ abundance (fluorescent intensity) of Cac and Liprin-α (***B***, *n* = 1,422 AZs from 6 NMJs from 6 larvae), Syd1 (***C***, *n* = 1,579 AZs from 6 NMJs from 6 larvae), RBP (***D***, *n* = 996 AZs from 6 NMJs from 6 larvae), and Unc13A (***E***, *n* = 984 AZs from 6 NMJs from 6 larvae) at third instar M4 NMJs. ***F*–*J***, Correlation between evoked *P_r_* and AZ abundance (fluorescent intensity) of Liprin-α (***F***, *n* = 913 AZs from 6 NMJs from 6 larvae), Syd 1 (***G***, *n* = 525 AZs from 6 NMJs from 6 larvae), RBP (***H***, *n* = 407 AZs from 6 NMJs from 6 larvae), Unc13A (***I***, *n* = 462 AZs from 8 NMJs from 6 larvae), and Cac (***J***, *n* = 486 AZs from 7 NMJs from 6 larvae) at third instar M4 NMJs. Raw values and statistical details are provided in Data S1.

Given sequential addition of proteins to maturing AZs, how their abundance correlates with synapse function was assayed. Presynaptic release strength has been previously correlated with AZ abundance of Ca^2+^ channels (Sheng et al., 2012; Akbergenova et al., 2018; Gratz et al., 2019; Medeiros et al., 2024), while late AZ scaffolding proteins like RBP and BRP facilitate clustering of Cac at *Drosophila* AZs (Kittel et al., 2006; Fouquet et al., 2009; Liu et al., 2011) and enhance release (Peled et al., 2014; Muhammad et al., 2015; Reddy-Alla et al., 2017; Akbergenova et al., 2018; Newman et al., 2022). Whether the abundance of early synaptic scaffolds regulates release output is unclear. To examine early versus late scaffold abundance and presynaptic output, fluorescently tagged lines expressing Liprin-α, Syd1, RBP and Unc13A were assayed for Cac levels and evoked *P_r_*. AZ Cac levels correlated weakly with the abundance of the early scaffolding proteins Liprin-α (Pearson's *r* value = 0.19) and Syd1 (Pearson's *r* value = 0.26, Fig. 5*A–C*). In contrast, AZ abundance of the late scaffolding proteins RBP (Pearson's *r* value = 0.51) and Unc13A (Pearson's *r* value = 0.66) were strongly correlated with Cac levels (Fig. 5*A*,*D*,*E*).

To assay AZ protein abundance and evoked release strength, functional imaging was performed in larvae expressing LexOP-myrGCaMP7s in muscles with *Mef2*-LexA and a UAS-fluorescently tagged AZ protein in MNs using *elav*-GAL4. As suggested by their low correlation with Cac abundance, AZ levels of Liprin-α (Pearson's *r* value = 0.13, Fig. 5*F*) and Syd1 (Pearson's *r* value = 0.12, Fig. 5*G*) poorly predicted evoked *P_r_* compared with the AZ abundance of RBP (Pearson's *r* value = 0.37, Fig. 5*H*) and Unc13A (Pearson's *r* value = 0.52, Fig. 5*I*). Even though late scaffolds correlated with evoked output, their abundance was slightly less predictive of *P_r_* that Cac AZ levels (Pearson's *r* value = 0.54, Fig. 5*J*). Although prior data indicate early AZ scaffolds regulate AZ seeding and synapse formation, our findings suggest their absolute abundance at mature release sites is less critical for Cac levels and *P_r_* than late-arriving AZ proteins like RBP and Unc13A that play a more direct role in SV and Ca^2+^ channel accumulation.

### Reduced synaptic activity increases AZ size via a Rab3-independent mechanism

**Figure 6.**
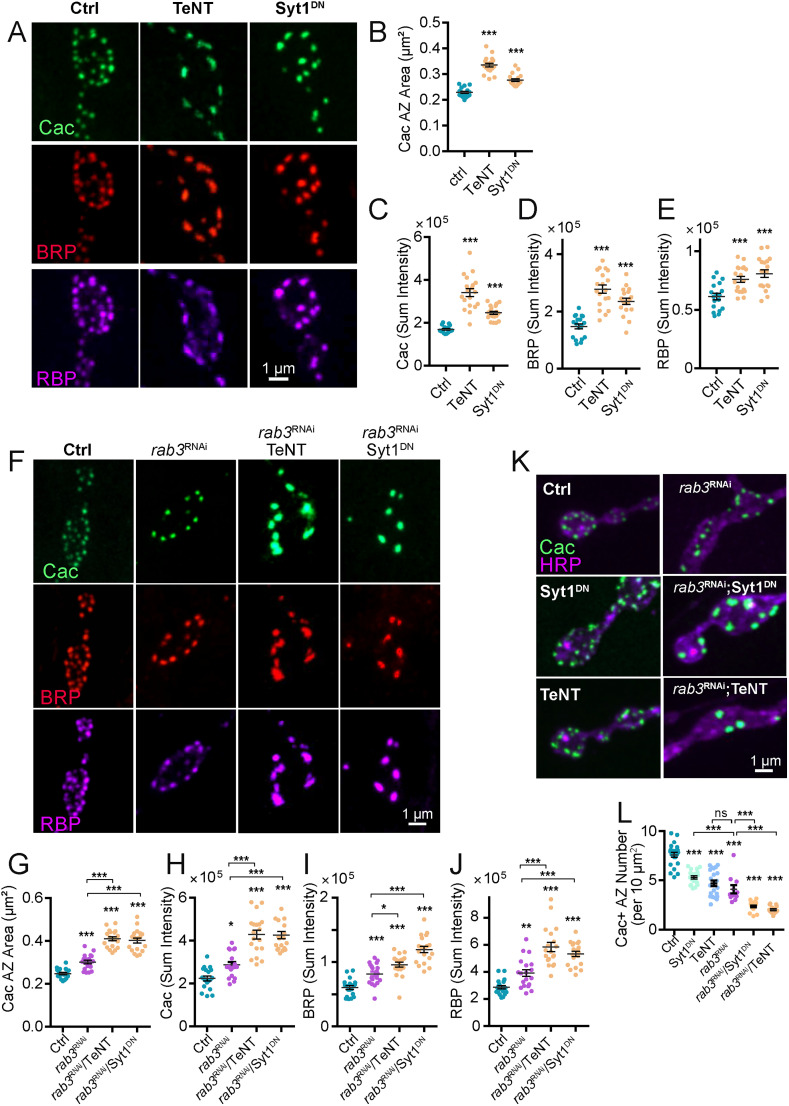
Presynaptic silencing results in AZ enlargement via a Rab3-independent mechanism. ***A***, Representative images of Cac, BRP, and RBP immunolabeling within synaptic boutons of third instar M1 NMJs of controls or following expression of TeNT (middle panel) or Syt1^DN^ (right panel) with the MN1-Ib-Gal4 driver. ***B***, Quantification of AZ Cac area in the three genotypes (Ctrl, *n* = 18 NMJs from 5 larvae; TeNT, *n* = 19 NMJs from 5 larvae; *p* < 0.0001; Syt1^DN^; *n* = 19 NMJs from 5 larvae; *p* < 0.0001). ***C–E***, Quantification of AZ sum Cac fluorescence (***C***, Ctrl, *n* = 18 NMJs from 5 larvae; TeNT, *n* = 19 NMJs from 5 larvae; *p* < 0.0001; Syt1^DN^, *n* = 19 NMJs from 5 larvae; *p* < 0.0001), BRP (***D***, Ctrl, *n* = 18 NMJs from 5 larvae; TeNT, *n* = 19 NMJs from 5 larvae; *p* < 0.0001; Syt1^DN^, *n* = 19 NMJs from 5 larvae; *p* < 0.0001) and RBP (***E***, Ctrl, *n* = 18 NMJs from 5 larvae; TeNT, *n* = 19 NMJs from 5 larvae; *p* < 0.0001; Syt1^DN^, *n* = 19 NMJs from 5 larvae; *p* < 0.0001) across the three genotypes. ***F***, Representative images of Cac, BRP, and RBP immunolabeling within synaptic boutons of third instar M1 NMJs of controls (left panel) or following expression of *rab3* RNAi alone (second panel) or coexpressed with TeNT (third panel) or Syt1^DN^ (right panel) with the MN1-Ib-Gal4 driver. ***G***, Quantification of AZ Cac area in the four genotypes (Ctrl, *n* = 19 NMJs from 5 larvae; *rab3* RNAi, *n* = 19 NMJs from 5 larvae; *p* = 0.0002; *rab3* RNAi + TeNT, *n* = 19 NMJs from 5 larvae; *p* < 0.0001; *rab3* RNAi + Syt1^DN^, *n* = 19 NMJs from 5 larvae; *p* < 0.0001). ***H–J***, Quantification of AZ sum fluorescence for Cac (***H***, Ctrl, *n* = 19 NMJs from 5 larvae; *rab3* RNAi, *n* = 19 NMJs from 5 larvae, *p* = 0.019; *rab3* RNAi + TeNT, *n* = 19 NMJs from 5 larvae; *p* < 0.0001; *rab3* RNAi + Syt1^DN^, *n* = 19 NMJs from 5 larvae; *p* < 0.0001), BRP (***I***, Ctrl, *n* = 19 NMJs from 5 larvae; *rab3* RNAi, *n* = 19 NMJs from 5 larvae; *p* = 0.0009; *rab3* RNAi + TeNT, *n* = 19 NMJs from 5 larvae; *p* < 0.0001; *rab3* RNAi + Syt1^DN^, *n* = 19 NMJs from 5 larvae; *p* < 0.0001), and RBP (***J***, Ctrl, *n* = 19 NMJs from 5 larvae; *rab3* RNAi, *n* = 19 NMJs from 5 larvae; *p* = 0.0009; *rab3* RNAi + TeNT, *n* = 19 NMJs from 5 larvae; *p* < 0.0001; *rab3* RNAi + Syt1^DN^, *n* = 19 NMJs from 5 larvae; *p* < 0.0001) across the four genotypes. ***K***, Representative images of Cac and HRP immunolabeling within synaptic boutons of M1 NMJs of controls or following expression of *rab3* RNAi, Syt1^DN^ or TeNT alone, or *rab3* RNAi coexpressed with TeNT or Syt1^DN^, with the MN1-Ib-Gal4 driver. ***L***, Quantification of Cac^+^ AZ number normalized to NMJ HRP area across the six genotypes (Ctrl, *n* = 22 NMJs from 5 larvae; *rab3* RNAi, *n* = 11 NMJs from 4 larvae, *p* < 0.0001; TeNT, *n* = 21 NMJs from 5 larvae; *p* < 0.0001; Syt1^DN^, *n* = 19 NMJs from 5 larvae; *p* < 0.0001; *rab3* RNAi + TeNT, *n* = 15 NMJs from 4 larvae; *p* < 0.0001; *rab3* RNAi + Syt1^DN^, *n* = 24 NMJs from 5 larvae; *p* < 0.0001). Statistical significance determined with one-way ANOVA followed by Tukey's multiple-comparison test. Asterisks denote the following *p* values: *p ≤ 0.05; **p ≤ 0.01; ***p ≤ 0.001. Raw values and statistical details are provided in Data S1.

Alterations in synaptic output can occur through both Hebbian and homeostatic mechanisms in response to changes in neuronal activity. At *Drosophila* NMJs, the segregation of GluRs within a PSD (stronger GluRIIA^+^-containing receptors at the center vs weaker GluRIIB^+^ at the periphery) is enhanced during early stages of synapse formation following elevated neuronal activity (Akbergenova et al., 2018). How changes in neuronal activity influence presynaptic AZ maturation and structure is less clear, though recent studies suggest AZ remodeling can contribute to homeostatic plasticity (Frank et al., 2020). To assay changes in AZ formation and maturation in response to altered neuronal activity, AZ protein area and accumulation were examined following manipulations that decrease synaptic output. Synaptotagmin 1 (Syt1) functions as the Ca^2+^ sensor for synchronous SV fusion and *syt1^−/−^* null mutants display a severe reduction in evoked release (Yoshihara and Littleton, 2002). Pan-neuronal expression of a dominant-negative *Syt1* transgene that blocks Ca^2+^ binding by the C2B domain (UAS-Syt1C2B^D416N,D418N^, hereafter as Syt1^DN^) causes lethality and reduces evoked release to a greater extent than observed in *syt1^−/−^* null mutants (Guan et al., 2017). Similarly, pan-neuronal expression of tetanus toxin light chain (hereafter referred to as UAS-TeNT) cleaves the v-SNARE n-Synaptobrevin (nSyb), causing lethality and abolishing evoked release (Sweeney et al., 1995). To characterize changes in AZ development following disruption to synaptic transmission, single neuron manipulations of synaptic output were performed using a MN1-Ib specific Gal4 driver that expresses in one of the ∼35 larval MNs that uniquely innervates M1 (Aponte-Santiago et al., 2020; Jetti et al., 2023). This approach bypasses confounding effects of whole-animal activity manipulations by restricting synaptic dysfunction to a single MN in each larval hemi-segment. TeNT or Syt1^DN^ were expressed in MN1-Ib and the distribution of BRP, RBP and Cac was analyzed and compared with control NMJs. Analysis of Cac AZ area revealed a 47% enlargement following TeNT expression and a 21% increase following Syt1^DN^ expression (Fig. 6*A*,*B*). In addition, AZ accumulation of BRP, RBP and Cac were enhanced at MN1-Ib NMJs expressing either TeNT or Syt1^DN^ (Fig. 6*C–E*). These data indicate Cac AZ size and the abundance of late AZ scaffolds and Cac channels are enhanced following a reduction in presynaptic release.

We next examined how changes in AZ size due to activity silencing compared with those previously identified in *Drosophila rab3* mutants. Loss of Rab3 cause a striking defect in AZ material accumulation, with Cac channels and late scaffolds hyperaccumulating at a subset of AZs, while other AZs are devoid of these components and only contain early arriving AZ proteins (Graf et al., 2009; Goel et al., 2019). To determine if the increase in AZ size following activity reduction requires the Rab3 pathway, UAS-*rab3* RNAi constructs were coexpressed in MN1-Ib with TeNT or Syt1^DN^. Expression of *rab3* RNAi alone induced synaptic defects at M1 NMJs similar to *rab3* mutants, including increased Cac AZ area (Fig. 6*F*,*G*), enhanced BRP, RBP and Cac levels at these AZs (Fig. 6*H–J*), and decreased BRP^+^ AZ density (Fig. 6*L*). Expression of TeNT or Syt1^DN^ together with *rab3* RNAi led to a synergistic enlargement of Cac AZ area (Fig. 6*F*,*G*) and even higher levels of BRP, RBP and Cac accumulation compared with controls or expression of TeNT, Syt1^DN^ or *rab3* RNAi alone (Fig. 6*H–J*). Beyond the increase in Cac AZ size and protein content, expression of TeNT or Syt1^DN^ at M1 NMJs also resulted in a reduction in Cac^+^ AZ number (Fig. 6*L*), indicating neuronal silencing decreases AZ seeding. Co-expression of *rab3* RNAi with TeNT or Syt1^DN^ resulted in a synergistic decrease in AZ density as well (Fig. 6*L*).

**Figure 7.**
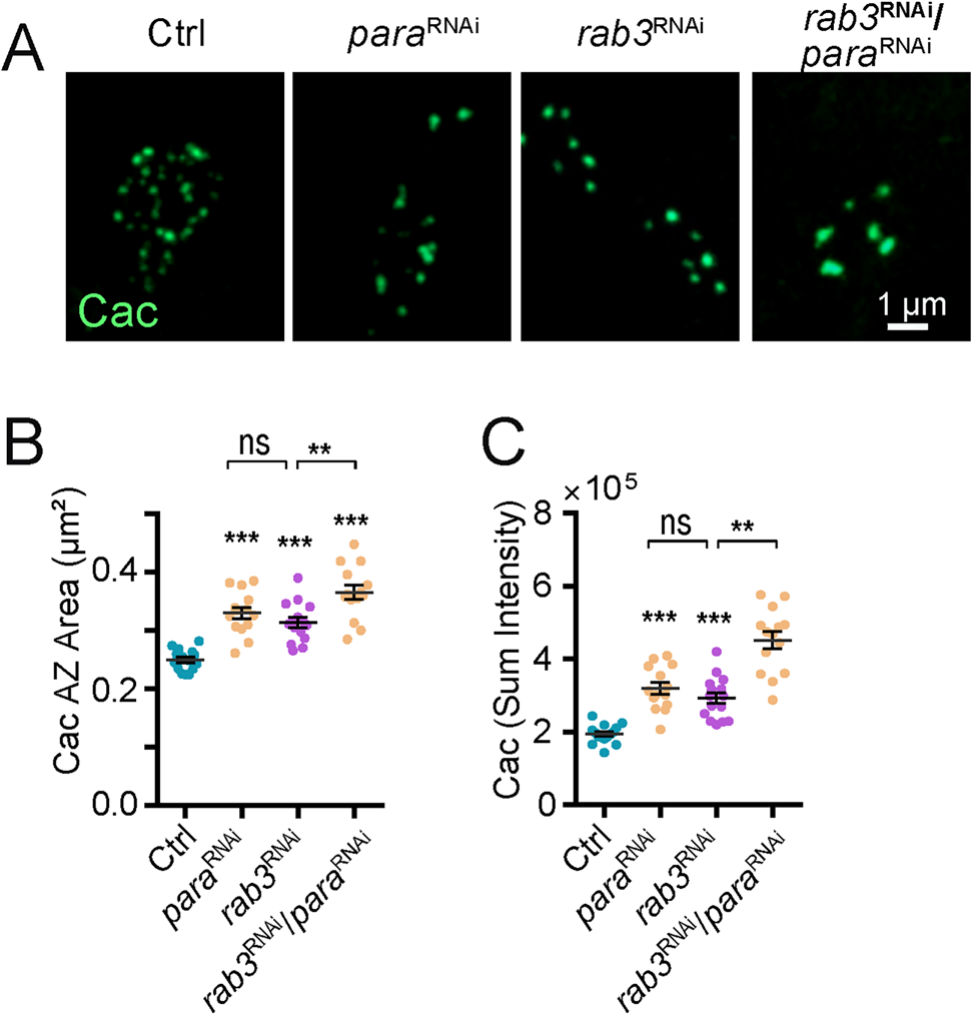
Presynaptic knockdown of the Para sodium channel mimics effects of blocking SV fusion on AZ enlargement. ***A***, Representative images of Cac immunolabeling within synaptic boutons of third instar M1 NMJs of controls (left panel) or following expression of *para* RNAi (second panel), *rab3* RNAi (third panel), or *para* RNAi + *rab3* RNAi (right panel) with the MN1-Ib-Gal4 driver. RNAi-mediated suppression of para also triggers AZ enlargement that is enhanced following Rab3 knockdown. Scale bar, 1 µm. ***B***, Quantification of the M1 AZ Cac area in the four genotypes (Ctrl, *n* = 15 NMJs from 4 larvae; *para* RNAi, *n* = 14 NMJs from 4 larvae; *p* < 0.0001; *rab3* RNAi, *n* = 15 NMJs from 4 larvae; *p* < 0.0001; *para* RNAi + *rab3* RNAi, *n* = 14 NMJs from 4 larvae; *p* < 0.0001). ***C***, Quantification of M1 AZ Cac sum fluorescence in the four genotypes (Ctrl, *n* = 15 NMJs from 4 larvae; *para* RNAi, *n* = 14 NMJs from 4 larvae; *p* < 0.0001; *rab3* RNAi, *n* = 15 NMJs from 4 larvae; *p* = 0.0002; *para* RNAi + *rab3* RNAi, *n* = 14 NMJs from 4 larvae; *p* < 0.0001). Statistical significance determined with Student's *t* test; asterisks denote the following *p* value: ***p ≤ 0.001. Raw values and statistical details are provided in Data S1.

To reduce synaptic activity independent of a blockage in SV fusion by TeNT or Syt1^DN^, UAS-RNAi knockdown of the Para voltage-gated Na^+^ channel was performed using the MN1-Ib-Gal4 driver. Like TeNT and Syt1^DN^, blocking action-potential generation in MN1-Ib increased AZ Cac area and Cac accumulation (Fig. 7*A–C*). These effects were also enhanced by co-expression of *rab3* RNAi. Given loss of Para disrupts action-potential–triggered evoked release and not spontaneous fusion, the effects of activity reduction on AZ maturation and size are likely downstream of signaling pathways associated with evoked output. Consistent with loss of evoked release being the major driver for AZ size expansion, the Syt1^DN^ transgene dramatically reduces evoked output while increasing the spontaneous release rate (Guan et al., 2017). Together, these data indicate synaptic inactivity and loss of Rab3 trigger AZ enlargement via distinct mechanisms, with neither pathway alone reaching an upper limit for these processes. We hypothesize neurotransmitter release at individual AZs activates a feedback loop to regulate synapse size and accumulation of synaptic components. At later stages when *P_r_* is established, a negative feedback signal arrests further AZ enlargement and material accumulation. When synaptic activity is reduced, negative feedback is suppressed, and AZs continue to accumulate material and increase in size.

### Distinct effects on AZ seeding in *rab3* mutants compared with activity silencing

**Figure 8.**
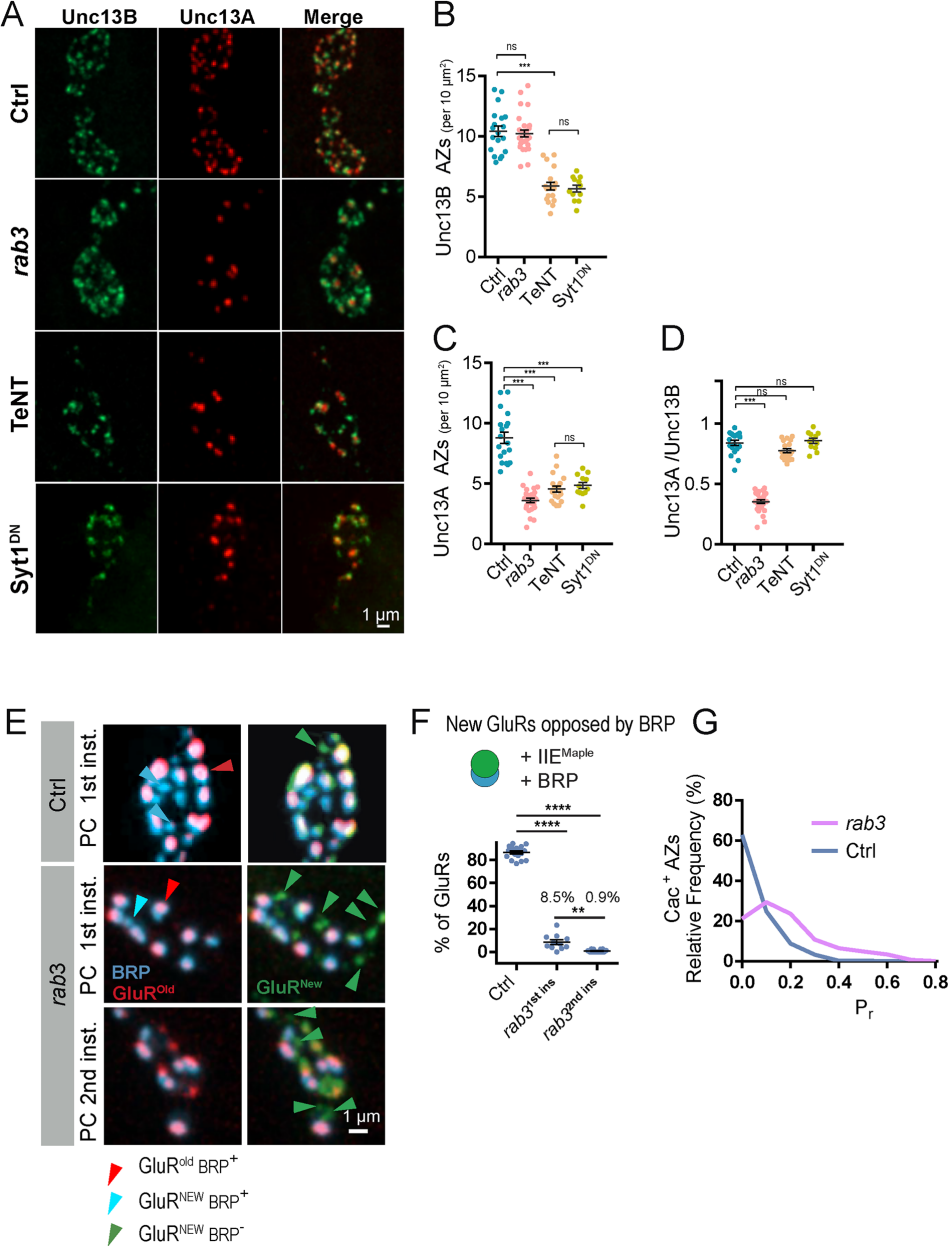
Presynaptic silencing reduces seeding of both early and late AZ scaffolds. ***A***, Representative images of Unc13B-mClover and Unc13A-mRuby localization within synaptic boutons of third instar M1 NMJs of controls (top panel) or following expression of *rab3* RNAi (second panel), TeNT (third panel), or Syt1^DN^ (bottom panel) with the MN1-Ib-Gal4 driver. The merged image is shown on the right. Scale bar, 1 µm. ***B***, Quantification of Unc13B^+^ AZ number (per 10 mm^2^) across the four genotypes (Ctrl, *n* = 19 NMJs from 5 larvae; *rab3* RNAi, *n* = 28 NMJs from 6 larvae; *p* = 0.99; TeNT, *n* = 19 NMJs from 5 larvae; *p* < 0.0001; Syt1^DN^, *n* = 12 NMJs from 4 larvae; *p* < 0.0001). ***C***, Quantification of the Unc13A^+^ AZ number (per 10 mm^2^) across the four genotypes (Ctrl, *n* = 19 NMJs from 5 larvae; *rab3* RNAi, *n* = 28 NMJs from 6 larvae; *p* < 0.0001; TeNT, *n* = 19 NMJs from 5 larvae; *p* < 0.0001; Syt1^DN^, *n* = 12 NMJs from 4 larvae; *p* < 0.0001). ***D***, Quantification of the Unc13A^+^/Unc13B^+^ AZ ratio across the four genotypes (Ctrl, *n* = 19 NMJs from 5 larvae; *rab3* RNAi, *n* = 28 NMJs from 6 larvae; *p* < 0.0001; TeNT, *n* = 19 NMJs from 5 larvae; *p* = 0.07; Syt1^DN^, *n* = 12 NMJs from 4 larvae; *p* = 0.57). ***E***, Representative images of third instar M4 boutons immunostained for BRP (cyan) after PC of GluRIIE^Maple^ at the first or second instar stage in *rab3* mutants or the first instar stage in controls. BRP^+^ AZs were rarely observed at newly formed green^+^/red^−^ GluR^New^ PSDs (green arrowheads) compared with older red^+^ PSDs (red arrowheads) after PC at the first instar stage in *rab3* mutants. When PC was performed in the second instar stage in *rab3* mutants, only older red^+^ PSDs were opposed to AZs that were BRP^+^. AZs opposed to newly formed green^+^/red^−^ PSDs were BRP^−^. Controls showed a normal pattern of synapse maturation, with AZs opposed to older red^+^ PSDs containing BRP and 89% of newly formed green^+^/red^−^ PSDs opposed to AZs being BRP^+^. ***F***, Quantification of BRP^+^ AZs opposed to newly formed green^+^/red^−^ PSDs after PC in the first instar stage in controls and *rab3* mutants (Ctrl, 86.6 ± 1.3%; *n* = 18 NMJs from 3 larvae; *rab3*, 8.5 ± 2.0%; *n* = 11 NMJs from 3 larvae; *p* > 0.001) and after PC in the second instar stage in *rab3* mutants (0.9 ± 0.3%; *n* = 14 NMJs from 3 larvae). ***G***, Relative *P_r_* frequency distribution across the AZ population for *rab3* and control M4 NMJs reveals a right-shifted and more homogenous distribution in *rab3* mutants. Statistical significance determined with one-way ANOVA followed by Tukey's multiple-comparison test. Asterisks denote the following *p* values: *p ≤ 0.05; **p ≤ 0.01; ***p ≤ 0.001; and ****p ≤ 0.0001. Raw values and statistical details are provided in Data S1.

To examine how synaptic inactivity decreases AZ number, we used CRISPR to endogenously tag Unc13B and Unc13A splice variants in the same transgenic line to directly visualize early and late AZ scaffold distribution in vivo (Fig. 8*A*). The density of AZs containing the late scaffold protein Unc13A was reduced at third instar M1 NMJs in *rab3* mutants (2.4-fold) and larvae expressing either TeNT (1.9-fold) or Syt1^DN^ (1.8-fold) with the MN1-Ib driver (Fig. 8*A*,*C*). In contrast, the density of AZs containing the early scaffold Unc13B was similar between controls and *rab3* mutants, while expression of TeNT (1.8-fold) or Syt1^DN^ (1.8-fold) reduced Unc13B^+^ AZs to a similar extent to Unc13A^+^ sites (Fig. 8*A*–*D*). We conclude that unlike Rab3 that controls the distribution of late scaffolds across the AZ population, synaptic inactivity results in a global reduction in AZ seeding that affects early (Unc13B) and late (Unc13A, RBP, BRP, and Cac) AZ components.

One hypothesis for the *rab3* phenotype is that the earliest forming AZs require a distinct maturation mechanism coupled to axonal pathfinding and target recognition that is Rab3-independent. Addition of late scaffolds and Cac to AZs formed during subsequent NMJ expansion would then require Rab3 function. This model predicts only AZs formed in the embryo and first instar stage would contain late AZ material in *rab3* mutants and that evoked *P_r_* heterogeneity would be reduced given mature AZs would be more age-matched than controls. To test this hypothesis, GluRIIE^Maple^ was expressed in controls and *rab3* mutants, and PC was performed at different larval stages. Larvae were subsequently immunostained to determine the presence of BRP at third instar AZs (Fig. 8*E*,*F*). Control NMJs PC at the first instar stage and assayed at the third instar stage had 87% of GluR^New^ PSDs opposed by BRP-containing AZs (Fig. 8*F*), consistent with a delay in late scaffold accumulation at some newly formed synapses. In contrast, 99.9% of GluR^Old^ PSDs were opposed by BRP-containing AZs (Fig. 8*F*), indicating all mature release sites have accumulated BRP. In the case of *rab3* mutants, if BRP^+^ AZs were only opposed to GluR^Old^ PSDs, it would argue that BRP cannot accumulate at AZs formed later in development in *rab3* mutants. Consistent with this model, BRP colocalized with just 8.5% of opposed PSDs that only contained GluR^New^ in *rab3* mutants when PC was performed at the first instar stage, compared with 87% in controls (Fig. 8*E*,*F*). Larvae PC at the second instar stage to ensure labeling of only the later wave of new synapses revealed an even lower percentage (0.9%) of BRP^+^ AZs opposed to GluR^New^ PSDs in *rab3* mutants. Consistent with AZ age and material accumulation correlating with evoked *P_r_*, quantal imaging in *rab3* mutants demonstrated *P_r_* distribution was more homogenous and shifted toward higher *P_r_* than controls (Fig. 8*G*). These data indicate early formed AZs contain late scaffolds and Cac channels in *rab3* mutants, with AZs formed later in development requiring Rab3 to accumulate these proteins. The preferential effect on late AZ proteins in *rab3* mutants contrasts with activity silencing, which reduces the number of AZs containing both early and late scaffolds.

### Synaptic inactivity increases T-bar area while loss of Rab3 causes multiple T-bars per AZ

**Figure 9.**
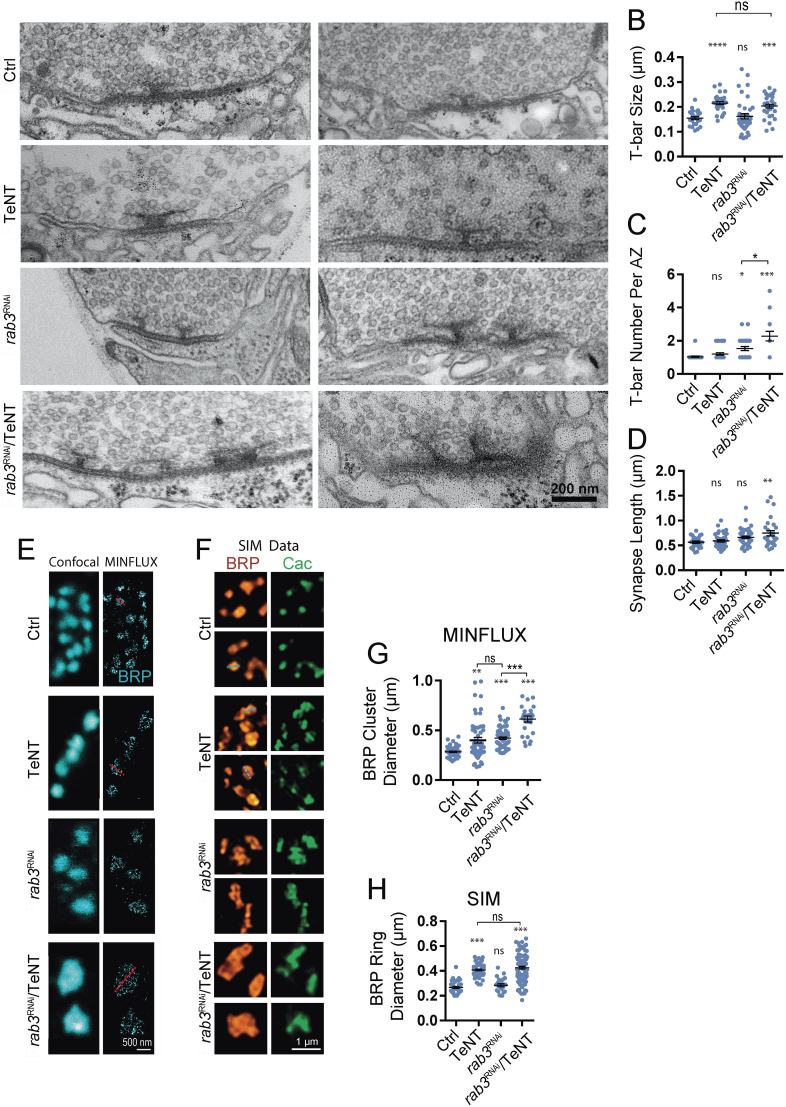
Presynaptic silencing increases T-bar area, while *rab3* mutants contain multiple T-bars per AZ. ***A***, Representative EM images of synaptic boutons at third instar M1 NMJs of controls (top panel) or following expression of TeNT (second panel), *rab3* RNAi (third panel), or *rab3* RNAi + TeNT (bottom panel) with the MN1-Ib-Gal4 driver. Scale bar, 200 nm. ***B***, Quantification of T-bar size (length of the top T-bar platform) across the four genotypes (Ctrl, *n* = 25 T-bars from 3 larvae; TeNT, *n* = 30 T-bars from 3 larvae; *p* < 0.0001; *rab3* RNAi, *n* = 39 T-bars from 3 larvae; *p* = 0.88; *rab3* RNAi + TeNT, *n* = 33 T-bars from 3 larvae; *p* = 0.0007). ***C***, Quantification of T-bar number per AZ across the four genotypes (Ctrl, *n* = 25 AZs from 3 larvae; TeNT, *n* = 29 AZs from 3 larvae; *p* = 0.74; *rab3* RNAi, *n* = 28 AZs from 3 larvae; *p* = 0.04; *rab3* RNAi + TeNT, *n* = 21 AZs from 3 larvae; *p* < 0.0001). ***D***, Quantification of synapse length (measurement of synaptic cleft electron-dense material length) across the four genotypes (Ctrl, *n* = 26 synapses from 3 larvae; TeNT, *n* = 37 synapses from 3 larvae; *p* = 0.90; *rab3* RNAi, *n* = 45 synapses from 3 larvae; *p* = 0.09; *rab3* RNAi + TeNT, *n* = 27 synapses from 3 larvae; *p* = 0.001). ***E***, Representative confocal and MINFLUX images of synaptic boutons at third instar M1 NMJs of controls (top panel) or following expression of TeNT (second panel), *rab3* RNAi (third panel), or *rab3* RNAi + TeNT (bottom panel) with the MN1-Ib-Gal4 driver. The red dashed line denotes a representative BRP cluster diameter measurement. Scale bar, 500 nm. ***F***, Representative SIM images of synaptic boutons at third instar M1 NMJs of controls (top panel) or following expression of TeNT (second panel), *rab3* RNAi (third panel), or *rab3* RNAi + TeNT (bottom panel) with the MN1-Ib-Gal4 driver. The blue dashed line denotes a representative BRP ring diameter measurement. Scale bar, 1 mm. ***G***, Quantification of BRP cluster diameter measured with MINFLUX across the four genotypes (Ctrl, *n* = 49 AZs from 3 larvae; TeNT, *n* = 53 AZs from 3 larvae; *p* = 0.0001; *rab3* RNAi, *n* = 65 AZs from 3 larvae; *p* < 0.0001; *rab3* RNAi + TeNT, *n* = 28 AZs from 3 larvae; *p* < 0.0001). ***H***, Quantification of BRP ring diameter measured with SIM across the four genotypes (Ctrl, *n* = 45 AZs from 3 larvae; TeNT, *n* = 59 AZs from 3 larvae; *p* < 0.0001; *rab3* RNAi, *n* = 24 AZs from 3 larvae; *p* = 0.71; *rab3* RNAi + TeNT, *n* = 95 AZs from 3 larvae; *p* < 0.0001). Statistical significance determined with one-way ANOVA followed by Tukey's multiple-comparison test. Asterisks denote the following *p* values: *p ≤ 0.05; **p ≤ 0.01; ***p ≤ 0.001. Raw values and statistical details are provided in Data S1.

Transmission electron microscopy (TEM) of *Drosophila* synapses demonstrated presynaptic AZs are highlighted by a central electron-dense body referred to as the T-bar (Atwood et al., 1993). Most AZs contain a single T-bar composed of scaffolding proteins like BRP (Fouquet et al., 2009; Wichmann and Sigrist, 2010), suggesting a modular organization for these release sites. To compare how loss of Rab3 and synaptic inactivation alter the ultrastructure of individual AZs, TEM was performed at larval M1 NMJs from controls or lines expressing TeNT, *rab3* RNAi, or both using the MN1-Ib driver (Fig. 9*A*). Silencing synaptic activity resulted in a 39% increase in the size of individual T-bars (Fig. 9*B*, measured as length of the top T-bar platform) but did not cause increased incorporation of T-bars at single AZs in MN1-Ib NMJs (Fig. 9*C*). Loss of Rab3 resulted in a distinct phenotype, with no change in the individual T-bar size but a ∼1.5-fold increase in the number of T-bars per AZ as previously reported (Graf et al., 2009). Reducing activity together with Rab3 depletion resulted in more severe AZ disorganization, with multiple enlarged T-bars per release site (Fig. 9*A*–*C*). In contrast to the increase in T-bar size or number, the length of the electron-dense synaptic cleft was largely unaffected, with only a mild enhancement following TeNT expression in *rab3* knockdown larvae (Fig. 9*D*).

To examine T-bar morphology at the nanoscale, MINFLUX imaging (Schmidt et al., 2021) that has a resolution comparable to EM was used, allowing visualization of filamentous BRP clusters at AZs (Fig. 9*E*). This single-molecule localization technology determines initially unknown fluorophore positions by relating it to the known zero-intensity position of a donut-shaped excitation beam. The diameter of BRP clusters increased following TeNT or *Rab3* RNAi expression and was further enlarged when TeNT and *Rab3* RNAi were coexpressed (Fig. 9*E*,*G*). Structural illumination microscopy (SIM) provided an optimal approach to image individual BRP AZ rings in larvae (Fig. 9*F*). Expression of TeNT resulted in a 53% enlargement in BRP ring diameter, while Rab3 reduction did not alter ring diameter but displayed multiple BRP rings at single AZs (Fig. 9*H*) as observed with TEM. When TeNT and *Rab3* RNAi were combined, larger BRP rings were present as clusters within individual AZs (Fig. 9*F*,*H*). These data indicate activity reduction increases the AZ size via a morphologically distinct process compared with loss of Rab3.

### AZ enlargement downstream of neuronal silencing is accompanied by changes in BRP turnover and requires postsynaptic GluRIIA-mediated signaling

**Figure 10.**
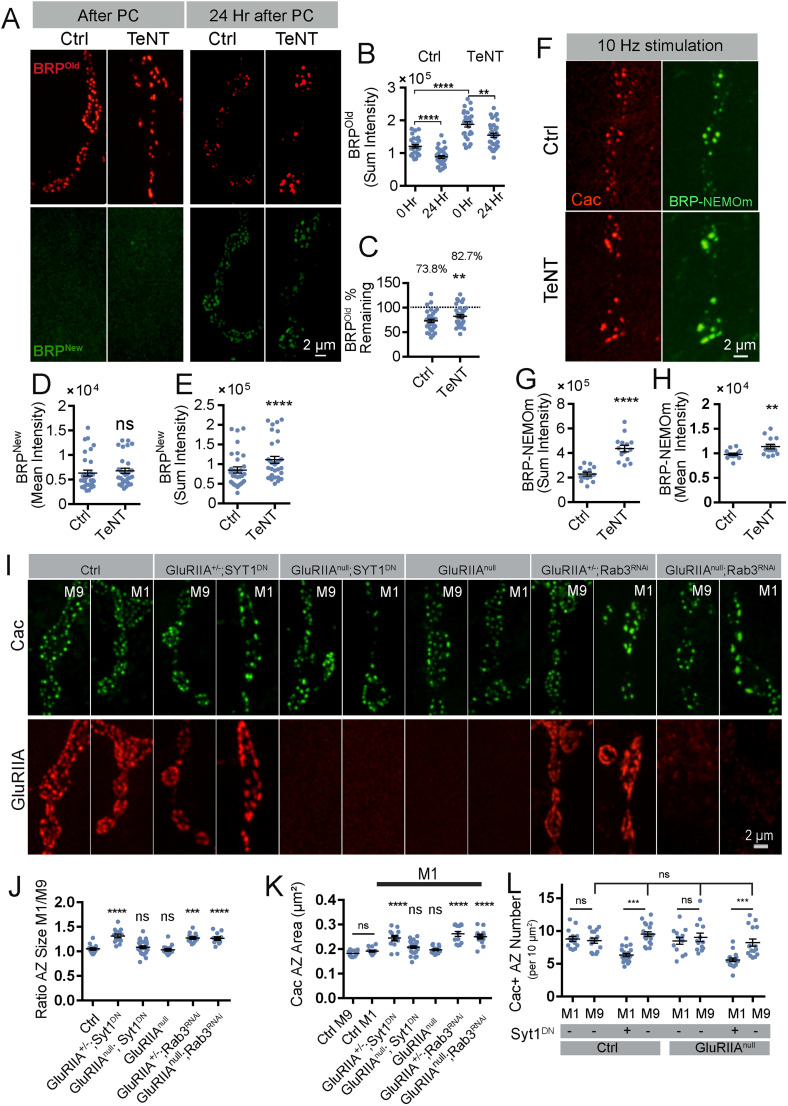
Changes in BRP turnover and role of GluRIIA in AZ enlargement following presynaptic silencing. ***A***, Representative images of mEosEM-tagged BRP before (green BRP^New^) and after (red BRP^Old^) PC of M9 (control) and M1 (TeNT) NMJs in larvae expressing UAS-TeNT with the MN1-Ib-Gal4 driver. ***B***, BRP^Old^ sum fluorescence per AZ puncta was compared before and 24 h after PC at M9 (control) and M1 (TeNT) NMJs (Ctrl 0 Hr, 121,279 ± 5,214; *n* = 28 NMJs from 7 larvae; Ctrl 24 Hr, 88,464 ± 4,428; *n* = 32 NMJs from 14 larvae; *p* < 0.0001; TeNT 0 Hr, 187,774 ± 7,831; *n* = 28 NMJs from 7 larvae; TeNT 24 Hr, 155,278 ± 6,958; *n* = 32 NMJs from 14 larvae; *p* = 0.0029). ***C***, Decay in BRP^Old^ as a percentage of the signal detected immediately after PC (Ctrl, 72.94% ± 3.65; *n* = 32 NMJs from 14 larvae; TeNT, 82.69 ± 3.71; *n* = 32 NMJs from 14 larvae; *p* = 0.0034). ***D***, Quantification of mean BRP^New^ fluorescence 24 h post-PC (normalized by pixel area of each AZ; Ctrl, 6,315 ± 613.4; *n* = 32 NMJs from 14 larvae; TeNT, 6,795 ± 533.8; *n* = 32 NMJs from 14 larvae; *p* = 0.0647). ***E***, Quantification of sum BRP^New^ fluorescence 24 h post-PC (Ctrl, 84,669 ± 7,899; *n* = 32 NMJs from 14 larvae; TeNT, 110,841 ± 8,890; *n* = 32 NMJs from 14 larvae; *p* < 0.0001). ***F***, Representative images showing BRP-NEMOm fluorescence in response to Ca^2+^ influx during 10 Hz stimulation in M9 (control) and M1 (TeNT) NMJs in larvae expressing UAS-TeNT with the MN1-Ib-Gal4 driver. Basal fluorescence of BRP-NEMOm without stimulation is undistinguishable from background (data not shown). ***G***, Quantification of sum BRP-NEMOm fluorescence per AZ (Ctrl, 229,046 ± 15,421; *n* = 14 NMJs from 7 larvae; TeNT, 437,306 ± 27,187; *n* = 14 NMJs from 7 larvae; *p* < 0.0001). ***H***, Quantification of mean BRP-NEMOm fluorescence per AZ (Ctrl, 9,784 ± 241.9; *n* = 14 NMJs from 7 larvae; TeNT, 11,362 ± 471.1; *n* = 14 NMJs from 7 larvae; *p* = 0.0044). Statistical significance determined with paired *t* test. ***I***, Representative images of Cac (top panel) and GluRIIA (bottom panel) immunolabeling at synaptic boutons of third instar M9 and M1 NMJs of controls, *GluRIIA^−/−^* null mutants or following expression of Syt1^DN^ or *rab3* RNAi with the MN1-Ib-Gal4 driver in control GluRIIA^+/−^ heterozygotes or GluRIIA^−/−^ nulls. ***J***, Quantification of the M1/M9 ratio for AZ Cac area across the six genotypes (Ctrl, *n* = 14 NMJs from 4 larvae; GluRIIA^+/−^ + Syt1^DN^, *n* = 14 NMJs from 4 larvae; *p* < 0.0001; GluRIIA^−/−^ + Syt1^DN^, *n* = 26 NMJs from 6 larvae; *p* = 0.84; GluRIIA^−/−^, *n* = 14 NMJs from 4 larvae; *p* = 0.83; GluRIIA^+/−^ + *rab3* RNAi, *n* = 13 NMJs from 4 larvae; *p* = 0.0002; GluRIIA^−/−^ + *rab3* RNAi, *n* = 14 NMJs from 4 larvae; *p* < 0.0001). ***K***, Quantification of AZ Cac area at control M1 and M9 NMJs and manipulated M1 NMJs across the five genotypes (Ctrl M9, *n* = 14 NMJs from 4 larvae; Ctrl M1, *n* = 14 NMJs from 4 larvae; GluRIIA^+/−^ + Syt1^DN^, *n* = 14 NMJs from 6 larvae; *p* < 0.0001; GluRIIA^−/−^ + Syt1^DN^, *n* = 26 NMJs from 4 larvae; *p* = 0.30; GluRIIA^−/−^, *n* = 14 NMJs from 4 larvae; *p* = 0.98; GluRIIA^+/−^ + *rab3* RNAi, *n* = 13 NMJs from 4 larvae; *p* < 0.0001; GluRIIA^−/−^ + *rab3* RNAi, *n* = 14 NMJs from 4 larvae; *p* < 0.0001). ***L***, Quantification of M1 and M9 Cac^+^ AZ number in controls or *GluRIIA^−/−^* null mutants with or without expression of Syt1^DN^ using the MN1-Ib-Gal4 driver (Ctrl M1, *n* = 14 NMJs from 4 larvae; Ctrl M9, *n* = 15 NMJs from 4 larvae; *p* = 0.99; M1 in MN1-Ib-Gal4 > Syt1^DN^, *n* = 20 NMJs from 5 larvae; *p* = 0.0003; M9 in MN1-Ib-Gal4 > Syt1^DN^, *n* = 19 NMJs from 5 larvae; *p* = 0.7; M1 in GluRIIA^−/−^, *n* = 13 NMJs from 4 larvae; *p* = 0.99; M9 in GluRIIA^−/−^, *n* = 13 NMJs from 4 larvae; *p* = 0.99; M1 in GluRIIA^−/−^ + MN1-Ib-Gal4 > Syt1^DN^, *n* = 17 NMJs from 4 larvae; *p* < 0.0001; M9 in GluRIIA^−/−^ + MN1-Ib-Gal4 > Syt1^DN^, *n* = 16 NMJs from 4 larvae; *p* = 0.90). Statistical significance determined with one-way ANOVA followed by Tukey's multiple-comparison test. Asterisks denote the following *p* values: *p ≤ 0.05; **p ≤ 0.01; ***p ≤ 0.001, and ****p ≤ 0.0001. Raw values and statistical details are provided in Data S1.

One hypothesis for enhanced AZ material accumulation and increased T-bar size is that silenced neurons increase transcription or translation of key AZ proteins to compensate for reduced synaptic output, resulting in increased transport and delivery of these proteins across development. Alternatively, the turnover of key AZ proteins could be reduced, leading to increased abundance and accumulation at release sites. To assay these potential mechanisms, an endogenously tagged photoconvertible BRP strain was generated by inserting mEosEM (Paez-Segala et al., 2015) into a previously described mimic site (Nagarkar-Jaiswal et al., 2015) within the *Brp* locus using CRISPR. mEosEM-tagged BRP localized to AZs as expected and could be completely and irreversibly photoconverted from green (hereafter referred to as BRP^New^) to red (hereafter referred to as BRP^Old^) using UV illumination through the cuticle (Fig. 10*A*) as previously described for GluR^Maple^. To determine if activity reduction altered BRP delivery or turnover, we expressed UAS-TeNT with MN1-Ib-GAL4 in the mEosEM-tagged BRP line. Following PC of silenced M1 NMJs and unaffected M9 NMJs in the same animal, BRP turnover was determined by comparing the rate of decay of red^+^ BRP^Old^ signal between the two NMJs 1 d after PC at 25°C (Fig. 10*B*,*C*). At control M9 NMJs, BRP^Old^ signal decreased by 27.1%, indicating a BRP half-life (T_1/2_) of 2.56 d. In contrast, the BRP^Old^ signal in M1-silenced NMJs in the same larvae decreased only 17.3%, suggesting a BRP T_1/2_ of 4.0 d and a ∼56% reduction in turnover within silenced synapses (Fig. 10*B*,*C*). To quantify BRP accumulation at AZs, we assayed the amount of newly delivered green^+^ BRP^New^ signal 24 h following PC (Fig. 10*A*,*D*,*E*). Silenced M1 NMJs displayed an increase in the sum intensity of BRP^New^ signal per AZ compared with unaffected M9 NMJs in the same larvae, consistent with expansion of the AZ area and more “slots” available for BRP seeding. However, the mean intensity of BRP^New^ per AZ was not affected, indicating BRP is spread over a larger AZ area. Given BRP^New^ will likely experience the same enhanced turnover rate during silencing, along with reduced AZ number under these conditions, this approach is poorly suited to precisely quantify BRP delivery rates. In conclusion, we find that neuronal silencing decreases BRP turnover, suggesting this mechanism is likely to contribute to enhanced BRP levels at AZs and the observed expansion in T-bar size.

The changes to AZs in silenced neurons suggest a compensation mechanism to increase synaptic output from individual release sites. Given SV fusion is blocked in silenced neurons expressing TeNT and Syt1^DN^, any compensation would fail to generate enhanced evoked output that could be measured electrophysiologically in the postsynaptic cell. However, these manipulations should not disrupt presynaptic Ca^2+^ influx from Cac channels given they target the SV fusion machinery directly. To determine if larger AZs in silenced neurons displayed enhanced function, we compared presynaptic Ca^2+^ influx between silenced and unaffected NMJs in the same animal. Transgenic lines containing the NEMOm Ca^2+^ indicator (Li et al., 2023) attached to the endogenous *Brp* locus at the N-terminus of the protein that resides near Cac channels were generated using CRISPR. Simulation of MNs at 10 Hz allowed robust detection of presynaptic Ca^2+^ influx that remained confined to single AZs (Movie 2; Fig. 10*F*). Consistent with enhanced function and increased Cac channels at larger AZs in silenced MNs, TeNT expression in MN1-Ib resulted in a 90.9% increase in presynaptic Ca^2+^ influx per AZ compared with control M9 NMJs in the same animal (Fig. 10*G*,*H*).

**Movie 2.** Imaging of presynaptic Ca^2+^ influx at a M1 NMJ in a third instar larva with NEMOm-BRP during 10 Hz stimulation. The NMJ area is outlined. During stimulation, the muscle contracts and stabilizes in the contracted position. The AZ fluorescence of NEMOm-BRP increases during stimulation before reaching a peak and stabilizing. Upon cessation of stimulation, NEMOm-BRP fluorescence decays to background levels as Ca^2+^ levels fall. [View online]

Changes to AZ structure have also been observed during presynaptic homeostatic plasticity (PHP) that contributes to enhanced SV release that helps maintain normal levels of synaptic output when postsynaptic GluR function is reduced (Weyhersmüller et al., 2011; Böhme et al., 2019; Mrestani et al., 2021). To examine if reduced presynaptic output may utilize a similar pathway as PHP downstream of loss or blockage of GluRIIA, we expressed Syt1^DN^ in MN1-Ib in *GluRIIA* null mutants (Han et al., 2023). In controls, the Cac AZ area was indistinguishable at M1 and neighboring M9 NMJs, resulting in a M1/M9 AZ area ratio of ∼1 (Fig. 10*I*,*J*). While expression of Syt1^DN^ in MN1-Ib triggered AZ enlargement at M1 NMJs, resulting in a larger M1/M9 AZ area in heterozygous *GluRIIA^+/−^* controls, expression in *GluRIIA* homozygous mutants failed to induce an enlargement in the AZ Cac area (Fig. 10*J*,*K*). In contrast, loss of GluRIIA did not prevent AZ size expansion in *rab3* mutants (Fig. 10*J*,*K*), consistent with distinct mechanisms mediating AZ enlargement downstream of Rab3. Although GluRIIA is necessary for AZ enlargement following reduced presynaptic output, it was not required for the corresponding reduction in AZ number induced by Syt1^DN^ expression (Fig. 10*L*). These data suggest increased AZ size is not strictly due to enhanced delivery of material over a reduced number of AZs following neuronal silencing. To determine if loss of GluRIIA alone resulted in an increase in AZ area that would preclude further expansion following Syt1^DN^ expression, AZ Cac area was compared between controls and *GluRIIA* null mutants. No increase in AZ Cac area was observed in *GluRIIA* mutants alone (Fig. 10*J*,*K*). Together, these data indicate postsynaptic GluRIIA-containing receptors are part of the mechanism for transducing reduced evoked release into retrograde signals that support increased presynaptic AZ size.

## Discussion

In the current study, we describe how AZ maturation in *Drosophila* larval MNs controls their output strength and how this process is regulated by neuronal activity. Using mMaple-tagged GluRs to timestamp individual synapses, we find that older AZs have a higher evoked *P_r_* than newly formed release sites. These data suggest AZs formed earlier in development have more time to accumulate scaffolding proteins and Ca^2+^ channels that promote higher *P_r_* compared with newly formed AZs. By time-stamping synapses in the first instar stage and recording their output in late third instars, early formed AZs also stably maintain their high *P_r_* state across the 6 d developmental window of larval development. Supporting a developmental AZ maturation model at larval NMJs, previous studies using serial intravital imaging identified sequential addition of early versus late AZ scaffolding proteins (Fouquet et al., 2009; Owald et al., 2010, 2012; Muhammad et al., 2015; Böhme et al., 2016; Ghelani and Sigrist, 2018). Using fixed and live imaging of a large panel of AZ proteins in the current study, Liprin-α is the first arriving AZ component detected, followed by the early scaffold proteins Syd1, RIM, and Unc13B. BRP, RBP, and Unc13A scaffolds appear later and around the same time, with Cac channels arriving several hours after RBP. In addition, the abundance of early AZ scaffolding proteins correlated poorly with Cac levels and evoked *P_r_* compared with late AZ scaffolds. Although our data support a time-dependent AZ maturation process, they don't eliminate the additional possibility that individual AZs are maturing to different evoked *P_r_* endpoints that are controlled by a yet unknown mechanism.

We also find that AZ maturation state regulates action-potential–independent spontaneous SV release. Prior studies revealed roles for spontaneous release in regulating synapse formation and synaptic plasticity at *Drosophila* NMJs (Yoshihara et al., 2005; Huntwork and Littleton, 2007; Korkut et al., 2013; Choi et al., 2014; Cho et al., 2015; Banerjee et al., 2021, 2024; Brija et al., 2023), highlighting a key function for this form of synaptic communication in neuronal development. Although AZs lacking Cac channels would not be expected to participate in evoked release, we observe many of these sites support spontaneous fusion. We propose “spontaneous-only” AZs at *Drosophila* NMJs (Melom et al., 2013) reflect an earlier stage of maturation prior to accumulation of sufficient Ca^2+^ channels to drive evoked fusion rather than a unique AZ state dedicated to this mode of synaptic communication. As such, AZ maturation regulates both release mode and output strength for individual synaptic sites. Although spontaneous-only AZs likely represent a developmental state at larval NMJs, there is ample evidence that this mode of release can be independently regulated from the evoked pathway to control synaptic growth (Cho et al., 2015; Mahoney et al., 2016; Brija et al., 2023).

How the sequential addition of AZs proteins is regulated in vivo within synaptic boutons is unclear. Prior evidence suggests early and late scaffolds can be assembled on a precursor vesicle from the trans-Golgi and transported together along axons (Maas et al., 2012; Vukoja et al., 2018; Götz et al., 2021; Rizalar et al., 2021). Our data suggest that if AZ components are codelivered in a common transport vesicle, they would need to be disassembled within synaptic boutons and sequentially added as AZs mature. Additionally, new AZs are continually forming at NMJs throughout development, with a ∼1.7-fold increase in AZ number each day during early larval stages (Akbergenova et al., 2018). This rapid addition of new release sites suggests the time course for AZ maturation could vary across development. Only a small number of AZs (∼8–10) are initially formed at embryonic NMJs, and we hypothesize enough material and assembly machinery are available for these sites to mature more quickly. When many more AZs are being added, shortages of material and/or regulatory components to rapidly assemble this large AZ cohort could delay the maturation rate. We hypothesize this mechanism accounts for the large population of low *P_r_* AZs observed at the late third instar stage when many newly added sites have not had sufficient time to fully mature. Given larval MNs burst fire at 20–30 Hz (Ormerod et al., 2022), having high *P_r_* AZs undergoing depression and low *P_r_* AZs undergoing facilitation is likely beneficial for providing a mechanism to maintain release output across the AZ population during high-frequency firing (Newman et al., 2017).

Neuronal activity provides an additional mechanism to regulate synapse maturation. Indeed, prior studies found elevated neuronal activity at *Drosophila* NMJs can enhance synaptic growth (Budnik et al., 1990; Guan et al., 2005; Mosca et al., 2005; Piccioli and Littleton, 2014) and promote faster maturation of postsynaptic GluR fields (Marrus and DiAntonio, 2004; Schmid et al., 2008; Petzoldt et al., 2014; Akbergenova et al., 2018; Zhao et al., 2020). In the current study, disrupting SV fusion with TeNT or Syt1^DN^ and blocking action potentials by knocking down the voltage-gated Na^+^ channel Para resulted in an enlargement in the AZ size and enhanced AZ protein content. Although the mechanisms for how loss of synaptic activity drives changes in AZ structure will require more analysis, we observed decreased BRP protein turnover at silenced NMJs. As such, AZ protein degradation is likely modulated by neuronal activity levels and contributes to the structural changes at presynaptic release sites.

The expansion in AZ size was also accompanied by a reduction in AZ number, indicating presynaptic silencing decreases seeding of new AZs. The mechanisms by which silenced presynaptic neurons control AZ size versus AZ addition are likely to be executed by distinct molecular programs, as *GluRIIA* mutants suppressed AZ expansion but did not prevent the reduction in AZ number. We hypothesize AZ expansion requires the postsynaptic muscle to report reduced neurotransmission through a GluRIIA-mediated retrograde signal, while AZ seeding may represent a cell autonomous mechanism or function via a GluRIIA-independent retrograde pathway. We observed a strong synergistic effect on increased AZ size and protein accumulation between the Rab3-dependent pathway and neuronal silencing, suggesting these represent independent mechanisms for controlling AZ size and protein content. Although it is unclear how Rab3 regulates accumulation of late scaffolds, it may be linked to its role as a regulator of vesicle trafficking and represent a failure in material delivery. Rather than accumulating at newly formed AZs, late scaffolds and Cac channels would be inserted at preexisting sites. Indeed, EM analysis revealed multiple normal size T-bars per AZ in *rab3* mutants compared with increased T-bar diameter in silenced neurons. Using CRISPR-tagging of the two splicing isoforms of Unc13, we found *rab3* mutants and neuronal silencing also decreased the number of functional release sites through independent mechanisms. While *rab3* mutants disrupt AZ formation at later stages (reduced Unc13A^+^ AZs), the ability to seed AZs containing early scaffolds remained intact (normal Unc13B^+^ AZ number). In contrast, neuronal silencing reduced the number of both Unc13A^+^ and Unc13B^+^ AZs, indicating a mechanism that targets the earliest steps of AZ seeding. We hypothesize that initial AZ seeding does not require Rab3-dependent trafficking steps, while neuronal silencing reduces expression or function of key AZ seeding factors.

**Figure 11.**
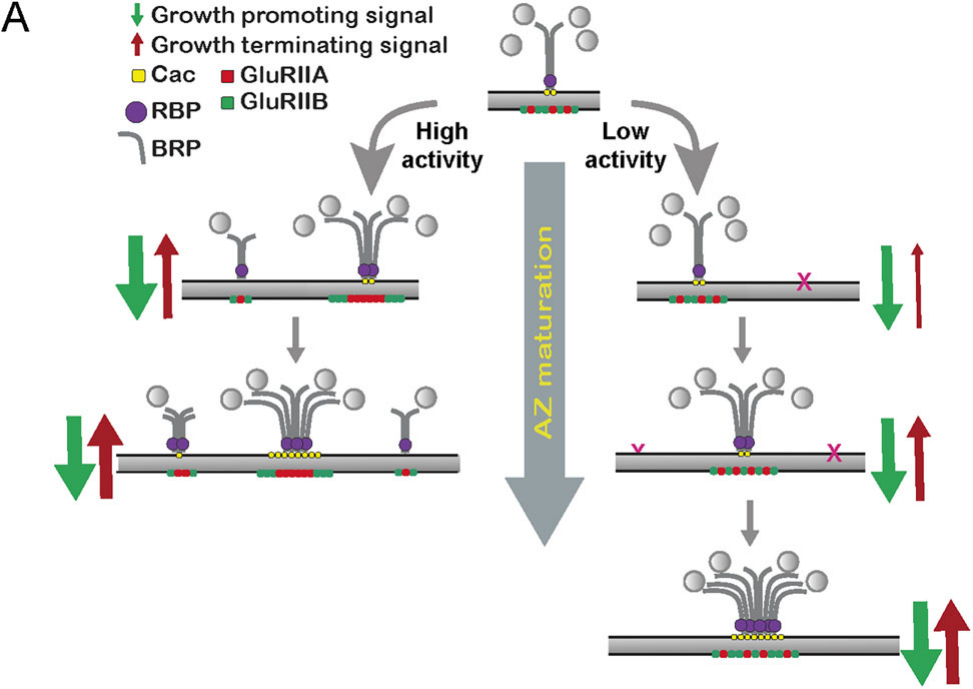
Model for the role of presynaptic output in synapse maturation. Under high activity conditions, both the AZ and PSD maturation rate is enhanced, and more synapses are seeded. Once AZs reach their normal high *P_r_* state, a maturation terminating signal requiring postsynaptic GluRIIA inhibits further AZ enlargement. Under conditions where presynaptic output is dramatically reduced, fewer synapses form. The reduction in presynaptic output disrupts the normal maturation stop signal, resulting in larger AZs containing more Cac and scaffolding proteins.

The effects of activity on synapse maturation are not limited to *Drosophila*, as silencing of activity in hippocampal neurons alters the nanoscale structure of the AZ matrix downstream of changes in the local actin cytoskeleton, resulting in enrichment of AZ components including Ca^2+^ channels (Glebov et al., 2017). Hippocampal silencing also increases AZ area and the number of docked SVs (Murthy et al., 2001). Enhancement in the levels of presynaptic Ca^2+^ channels in response to activity silencing has also been observed in mammalian cortical neuronal cultures (Lazarevic et al., 2011). Similar to our observations, activity deprivation in cortical neurons can increase presynaptic size and AZ protein abundance, together with a reduction in overall synapse number (Wise et al., 2024). These morphological changes are often tethered to homeostatic plasticity pathways that counteract chronic hypo- or hyper-activity states within neuronal circuits (Turrigiano and Nelson, 2004). Although the molecular pathways that regulate activity-dependent AZ seeding and maturation will require further work, our findings suggest a model for further experimental analysis (Fig. 11). In the early steps of AZ development, enhanced synaptic activity has a positive role in promoting AZ material accumulation and seeding of new AZs. Once an individual AZ reaches a preferred state of output, GluRIIA receptors in the opposed PSD facilitate transmission of a local retrograde signal that terminates further AZ expansion. In cases where presynaptic AZs are silenced, the signal to inhibit AZ expansion is reduced, resulting in larger AZs that contain more presynaptic material. In summary, our data indicate AZs require a developmental maturation process at *Drosophila* NMJs that controls both release mode and output strength. Alterations in neuronal activity alter AZ maturation and AZ number, with neuronal silencing resulting in expansion of AZ size with excess material accumulation.
